# Magnesium Uptake by CorA Transporters Is Essential for Growth, Development and Infection in the Rice Blast Fungus *Magnaporthe oryzae*

**DOI:** 10.1371/journal.pone.0159244

**Published:** 2016-07-14

**Authors:** Md. Hashim Reza, Hiral Shah, Johannes Manjrekar, Bharat B. Chattoo

**Affiliations:** Genome Research Centre, Department of Microbiology & Biotechnology Centre, Faculty of Science, The Maharaja Sayajirao University of Baroda, Vadodara 390 002, Gujarat, India; University of Nebraska-Lincoln, UNITED STATES

## Abstract

*Magnaporthe oryzae*, the causative organism of rice blast, infects cereal crops and grasses at various stages of plant development. A comprehensive understanding of its metabolism and the implications on pathogenesis is necessary for countering this devastating crop disease. We present the role of the CorA magnesium transporters, MoAlr2 and MoMnr2, in development and pathogenicity of *M*. *oryzae*. The *MoALR2* and *MoMNR2* genes individually complement the Mg^2+^ uptake defects of a *S*. *cerevisiae* CorA transporter double mutant. *MoALR2* and *MoMNR2* respond to extracellular Mg^2+^ and Ca^2+^ levels and their expression is elevated under Mg^2+^ scarce conditions. RNA silencing mediated knockdown of *MoALR2* (WT+siALR2, *Δmnr2*+siALR2 and *ALR2*+*MNR2* simultaneous silencing) drastically alters intracellular cation concentrations and sensitivity to metal ions. *MoALR2* silencing is detrimental to vegetative growth and surface hydrophobicity of mycelia, and the transformants display loss of cell wall integrity. *MoALR2* is required for conidiogenesis and appressorium development, and is essential for infection. Investigation of knockdown transformants reveal low cAMP levels and altered expression of genes encoding proteins involved in MoMps1 cell wall integrity and cAMP MoPmk1 driven MAP Kinase signaling pathways. In contrast to *MoALR2* knockdowns, the *MoMNR2* deletion (*Δmnr2*) shows increased sensitivity to CorA inhibitors as well as altered cation sensitivity, but has limited effect on surface hydrophobicity and severity of plant infection. Interestingly, *MoALR2* expression is elevated in *Δmnr2*. Impairment of development and infectivity of knockdown transformants and altered intracellular cation composition suggest that CorA transporters are essential for Mg^2+^ homeostasis within the cell, and are crucial to maintaining normal gene expression associated with cell structure, signal transduction and surface hydrophobicity in *M*. *oryzae*. We suggest that CorA transporters, and especially *MoALR2*, constitute an attractive target for the development of antifungal agents against this pathogen.

## Introduction

Rice blast disease caused by *Magnaporthe oryzae* continues to be a serious and recurring problem in all rice growing regions across the world. The rice blast fungus attacks rice plants at all stages of development and can infect leaves, stems, nodes, panicles and roots. Foliar infection occurs by formation of a dome-shaped infection structure called the appressorium, which upon maturation generates turgor pressure by accumulating high concentrations of compatible solutes such as glycerol [1] and is important for breaching the rice cuticle; thereby the fungal hyphae invade and ramify through the plant tissue and grow within the host cells. The fungus sporulates profusely from disease lesions under conditions of high humidity, allowing the disease to spread rapidly to adjacent rice plants by wind and dewdrop splash [2]. Considering the poor durability of many blast-resistant cultivars of rice, which have a typical field life of only 2–3 growing seasons before disease resistance is overcome, and increasing energy costs which affect fungicide and fertilizer prices, there is a need for better understanding of rice blast disease to combat this deadly crop destroyer [3]. Rice blast control strategies that can be deployed as part of an environmentally sustainable plan for increasing the efficiency of cereal cultivation are therefore urgently required [4].

The development of spores leading to appressorium formation is initiated through recognition of environmental cues and is mediated by cross-talk between signal transduction pathways within the cell. In the past two decades, studies on signaling pathways, which include Mitogen Activated Protein Kinase (MAPK) signaling cascade and signaling pathways dependent on secondary messengers like Ca^2+^ [5] and cAMP [6, 7], which regulate various stages of the *M*. *oryzae* infection cycle, have been initiated. Although the cell cycle and signal transduction pathways tightly regulate *M*. *oryzae* development and infection, studies of how metal ions affect these developmental pathways have been largely limited to calcium signaling. The ability to grow, divide, respond to cell wall stress, sporulate and infect are complex but critical processes in *M*. *oryzae* for its colonization and establishment in the host as a successful pathogen. Magnesium being a co-factor for a wide range of enzymes is important in a variety of biochemical processes. Mg^2+^ is utilized by twice as many metalloenzymes as Zinc [8]. Free Mg^2+^ is essential for stabilizing cell membrane, cell wall [9–12] and ribosomes. It is essential for neutralizing the negatively charged phosphate groups of nucleic acids [13], DNA repair, and is indispensable for DNA replication fidelity. Mg^2+^ regulates electrolyte transport across the cell membrane [13], as well as activity of the sodium potassium pump (Na/K-ATPase) and the calcium pump (Ca-ATPase) [14]. In the fission yeast *Schizosaccharomyces pombe* and the budding yeast *Kluyveromyces fragilis*, intracellular levels of Mg^2+^ regulate the timing of cell cycle progression [15]. Among pathogens, Mg^2+^ is also required for germ tube formation in *Candida albicans* vegetative cells and consequently affects its morphogenesis and pathogenicity [16]. Regulation of intracellular concentration of Mg^2+^ is achieved by three mechanisms: uptake systems, efflux from the cell and sequestration within organelles [17]. However, the relation between Mg^2+^ concentrations and morphogenesis has not been investigated in fungal plant pathogens, including *M*. *oryzae*.

The molecular identity, function and regulation of Mg^2+^ transporters have been studied extensively to understand the basis of Mg^2+^ homeostasis in eukaryotic cells. The CorA (or Metal Ion Transporter) superfamily is an important group of Mg^2+^ transporters in both prokaryotes and eukaryotes [17]. Despite divergent primary protein sequence, the CorA Mg^2+^ transporters are characterized by two or three conserved transmembrane domains near the carboxy terminus, one of which is followed by the conserved motif (F/W) GMN [18] that is essential for Mg^2+^ transport. In *Salmonella typhimurium* and *Escherichia coli*, three proteins (CorA, MgtA, and MgtB) have been shown to be involved in Mg^2+^ transport across the plasma membrane [18]. Magnesium uptake by CorA is essential for viability of *Helicobacter pylori* [19]. Eukaryotic CorA proteins have diversified in function, facilitating both Mg^2+^ uptake and distribution between sub-cellular compartments. *Saccharomyces cerevisiae* Alr1 is the first characterized Mg^2+^ transport system in eukaryotes and is distantly related to the bacterial CorA Mg^2+^ transporter family [18]. Subsequently a second CorA protein, Alr2, was identified in *S*. *cerevisiae*. Alr1 and Alr2 are present on the plasma membrane; loss-of-function mutations in Alr1 result in reduced Mg^2+^ uptake and growth defects restorable by external Mg^2+^ supplementation [18, 20]. Alr2 makes only a minor contribution to Mg^2+^ homeostasis, due to low expression and activity [14]. The Alr1 clade of CorA proteins includes a subgroup represented by Mnr2, a vacuolar membrane protein required for access to intracellular magnesium stores [21]. Another subfamily includes the yeast Mrs2 protein, which supplies Mg^2+^ to the mitochondrial matrix [22]. In *Arabidopsis thaliana*, a family of 10 Mg^2+^ transporters which is homologous to the yeast *MRS2* gene and to the CorA family in bacteria has been identified, most of which have been shown to be expressed in a range of plant tissues [23].

Given its diverse metabolic roles, magnesium is indispensable for cellular functioning. However, the regulation and role of CorA Mg^2+^ transporters in development and pathogenicity of *M*. *oryzae* are still unexplored. Considering the diverse roles of Mg^2+^ ions, understanding the regulation of Mg^2+^ in *M*. *oryzae* is of considerable interest.

In the present study we identified the *M*. *oryzae* orthologs of *S*. *Cerevisiae ALR1*, Mgg_08843 (*MoALR2*) and Mgg_09884 (*MoMNR2*). Both *MoALR2* and *MoMNR2* can complement the Mg^2+^ uptake defects in a *S*. *cerevisiae alr1 alr2* double mutant. As a first step towards understanding Mg^2+^ regulation, we show that CorA transporters in *M*. *oryzae* affect intracellular Mg^2+^ concentration and are in turn themselves regulated by levels of extracellular Mg^2+^ and other divalent cations. Using knockout and knockdown transformants we show that Mg^2+^ uptake by CorA transporters is required for fungal development, progression of the infection cycle and cell wall integrity. We find that in the knockdown transformants cAMP levels are reduced and expression of genes involved in key signaling pathways is altered. We show that depletion of CorA transporters mimics the phenotypes produced by extracellular Mg^2+^ scarcity and brings about changes in gene expression previously not known to be affected by Mg^2+^ in fungal pathogens. Analysis of both the transporters indicates that the role of *MoALR2* is more critical than that of *MoMNR2*, and that *MoALR2* may be indispensible for the growth and pathogenesis in *M*. *oryzae*. Our results indicate that both *MoALR2* and *MoMNR2* play important roles in Mg^2+^ homeostasis in *M*. *oryzae*, in which Alr2 appears to be more central than Mnr2.

## Results

### Identification of *MoALR2* and *MoMNR2*

**Fig 1:**
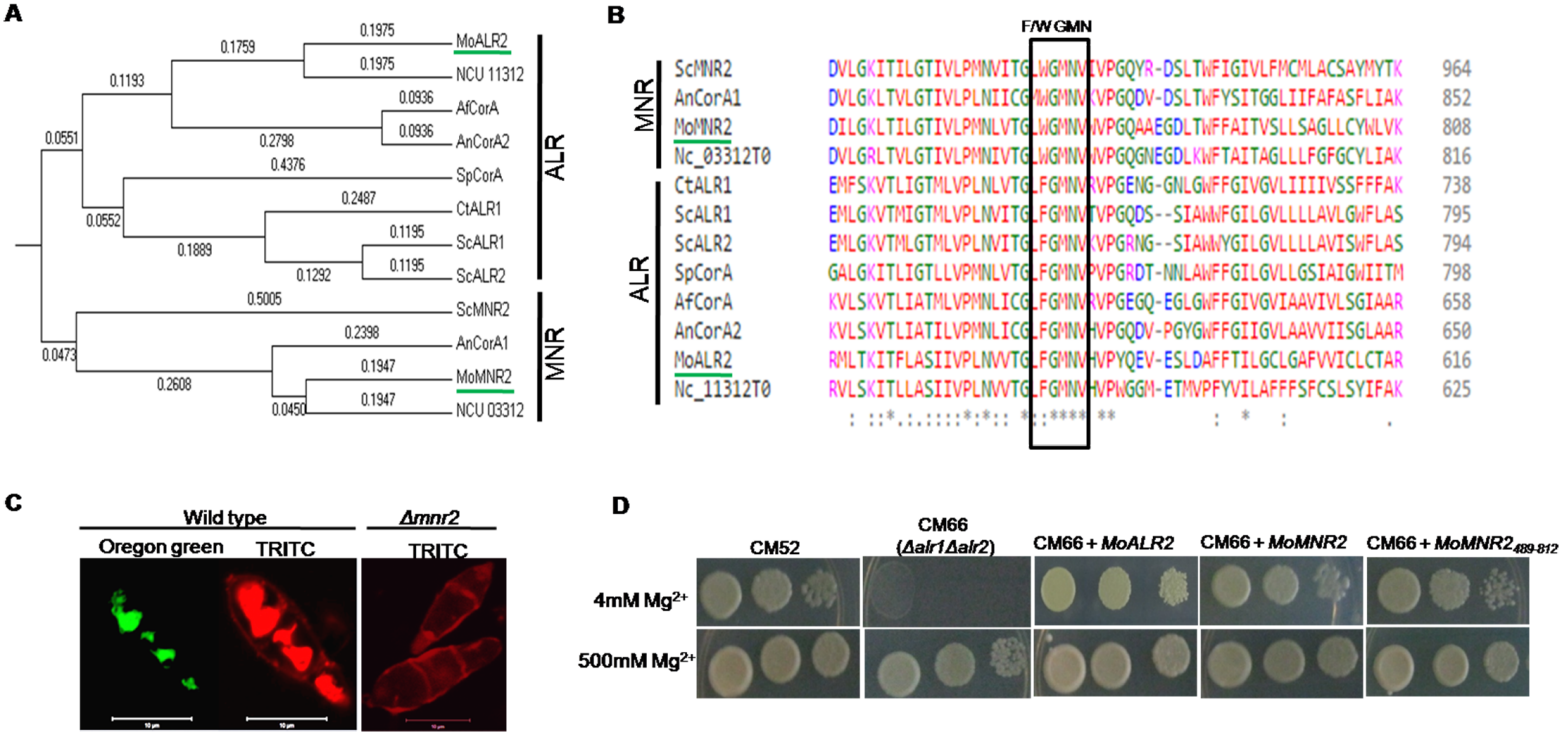
Identification of *MoALR2* and *MoMNR2*. (A)The tree was constructed using the Neighbor-Joining method based on alignment of full length sequences of CorA proteins of *Magnaporthe oryzae* (XP_003713862, XP_003709977), *Saccharomyces cerevisiae* (EDV10489, EDV09793, YKL064W), *Aspergillus fumigatus* (XP_754049), *Aspergillus nidulans* (CBF70700, CBF77902), *Candida tropicalis* (XP_002548119), *Schizosaccharmoyces pombe* (NP_595545) and *Neurospora crassa* (NCU11312, NCU03312). The evolutionary distances were computed using the Poisson correction method and are in the units of the number of amino acid substitutions per site. All positions containing gaps and missing data were eliminated. Evolutionary analyses were done using MEGA5. (B) Amino acid sequence alignment of CorA proteins *Magnaporthe oryzae* (XP_003713862, XP_003709977), *Saccharomyces cerevisiae* (EDV10489, EDV09793, and YKL064W), *Aspergillus fumigatus* (XP_754049), *Aspergillus nidulans* (CBF70700, CBF77902), *Candida tropicalis* (XP_002548119), *Schizosaccharmoyces pombe* (NP_595545) and *Neurospora crassa* (NCU11312, NCU03312) was performed using Clustal Omega. CorA consensus sequence motifs (Y/FGMN) for the above proteins are highlighted. (C) Conidia were harvested and treated with polyclonal antibodies raised against the CorA domain of MoMnr2. TRITC labeled secondary antibodies were used for staining. Oregon green 488 staining was used to visualize the vacuole. (D) *S*. *cerevisiaeΔalr1Δalr2* mutant (CM66) was transformed with pYES2-*MoALR2*, pYES2-*MoMNR2* and pYES2-*MoMNR2*_*489-812*_. The transformants were grown overnight on SD+Gal+leu+lys-ura+500mM Mg^2+^ and different dilutions were spotted on SD+Gal–ura+leu+lys containing 4mM Mg^2+^ and 500mM Mg^2+^ and grown at 28°C for 4 days. The experiments were repeated in triplicate, N = 3.

We identified CorA Magnesium transporters from the *M*. *oryzae* genome (http://www.broadinstitute.org/annotation/genome/magnaporthe_grisea/MultiHome.html) by a BLAST_P search using the full length *S*. *cerevisiae*Alr1proteinsequence (859 amino acids). We obtained two putative orthologs in the *M*. *oryzae* genome: Mgg_08843 (47% identity) and Mgg_09884 (49% identity), which are named MoAlr2 and MoMnr2 respectively. Both these proteins have two transmembrane domains towards the carboxy terminus, which are followed by conserved residues of (W/F) GMN, and hence belong to the CorA superfamily of Mg^2+^ transporters. MoAlr2 is a 622 amino acid protein with a CorA domain spanning amino acids 310–617 (Pfam). Strongly preferred model (ExPASy) for MoAlr2 predicts that the protein has two transmembrane helices (565–582; in-out) and (596–615; out-in), with the N-terminus facing the cytosol; the protein has been predicted to be localized to the plasma membrane (WoLFPSORT). MoMnr2is a 814 amino acid protein with a CorA domain spanning amino acids 491–809 (Pfam). MoMnr2 is predicted to have two transmembrane helices (756–773; in-out) and (788–806; out-in), with the N- terminus facing the cytosol. In *S*. *cerevisiae*, the ScMnr2 protein has been shown to be localized to vacuolar membrane. The MoAlr2 and MoMnr2 CorA domains have ~ 48% identity with that of the *S*. *cerevisiae* CorA domain. Multiple sequence alignment of CorA superfamily transporters across different species including yeast and several filamentous pathogenic and non-pathogenic fungi was done using full length protein sequence and the phylogenetic relationship of these CorA proteins is presented (Fig 1A). The CorA transporters included in the analysis form two clades. The MNR clade has W (tryptophan) just before the conserved sequence motif of GMN, while the ALR clade has F (phenylalanine) prior to the GMN sequence (Fig 1B).

CorA transporters are known to be present on the plasma membrane as well as on organelles to form a system of Mg^2+^ uptake and compartmentalization that maintains cytoplasmic Mg^2+^ homeostasis. In yeast, ScAlr2 has been shown to be a plasma membrane protein while ScMnr2 is a vacuolar membrane protein. The subcellular localization of CorA transporters in *M*. *oryzae* was done by indirect immunolocalization using polyclonal antibodies against the MoMnr2 CorA domain. In wild type, the CorA transporters localized to plasma membrane and vacuole (Fig 1C). Vacuolar localization was seen by co-localization with Oregon Green 488 staining (which stains vacuolar lumen). In the *MoMNR2* knockout (*Δmnr2*), the MoAlr2 protein was found to be restricted to the plasma membrane (Fig 1C).

To confirm the nature of these Mg^2+^ transporters functionally, complementation with the *M*. *oryzae* genes was carried out in yeast. The *S*. *cerevisiae Δalr1Δalr2* mutant CM66 [23] is a haploid disruptant for both *ALR1* and *ALR2* genes. Unlike the wild type, the double mutant is unable to grow at 4mM Mg^2+^, indicating a defect in Mg^2+^ uptake. To test the ability of *MoALR2* and *MoMNR2* to complement the Mg^2+^ uptake defect in the double mutant CM66, transformants over-expressing either *MoALR2* or *MoMNR2*, were first grown in SD media containing 500mM Mg^2+^ and then different dilutions were spotted on SD media containing 4mM Mg^2+^. The transformants were able to grow even at 4mM Mg^2+^ like the wild type, while the mutant could not (Fig 1D), suggesting that both *MoALR2* and *MoMNR2* could rescue the Mg^2+^ uptake defect and hence have a role in Mg^2+^ transport. Further, a truncated *MoMNR2* was also able to complement the function in the yeast mutant, suggesting that amino acids of the CorA domain at the Carboxyl terminus (489–812 amino acids) are sufficient for Mg^2+^ transport.

### CorA Mg^2+^ transporters alter metal ion composition in *M*. *oryzae*

Targeted disruption of *MoALR2* and *MoMNR2* through homologous recombination was attempted to investigate the functions of CorA Mg^2+^ transporters in *M*. *oryzae*. *MoMNR2* knockouts (*Δmnr2*) were generated using a Zeocin resistance cassette in the wild type strain B157 (WT). Three independent transformants were confirmed by Southern blotting for targeted gene deletion (S1B Fig). Immunostaining of *Δmnr2* with CorA antibodies showed staining only of plasma membrane due to MoAlr2, while vacuolar staining was absent (Fig 1C). In contrast, only non-homologous (ectopic) transformants were obtained for *MoALR2* despite multiple attempts using different conditions of selection (S1 Table). Knockout of *MoALR2* was also attempted in the *Δku80* background known to aid homologous integration. But screening of all the transformants resulted only in ectopic integrants (S1 Table). Thus, we speculate that *MoALR2* is essential for viability of *M*. *oryzae*.

To establish the role of *MoALR2* by an alternative approach, the gene was silenced in both WT and *Δmnr2* backgrounds using a stretch of 110bp complementary to the 5’ UTR of *MoALR2* [24], cloned in the vector pSD2. The knockdown was validated by analysis of relative expression of *MoALR2* and *MoMNR2* by quantitative Real Time PCR (qRT-PCR) in the transformants. Transformants in the WT background showed transcript levels of *MoALR2* ranging from 48% to 85%, while those in the background of *Δmnr2* showed transcript levels ranging from 66% to 88% compared to WT (S2 Table). No transcripts of *MoMNR2* were detected in *Δmnr2* background transformants, while the levels of *MoMNR2* did not change in the *MoALR2* knockdown transformants in wild type background, thereby confirming the specificity of the cassette used for *MoALR2* silencing. Since *MoALR2* could not be silenced more strongly in the *Δmnr2* background, to obtain transformants with further reduced transcript levels of *MoALR2*, an alternative knockdown approach for simultaneous silencing of both *MoALR2* and *MoMNR2* was also used. As these two genes show high similarity in the CorA domain, we carried out simultaneous silencing using an antisense construct targeted to this region. The knockdown was validated by qRT-PCR of *MoALR2* and *MoMNR2* in the transformants. The transformants showed transcript levels ranging from 30% to 80% and 37% to 90% for *MoALR2* and *MoMNR2* respectively, compared to WT (S2 Table).

**Fig 2:**
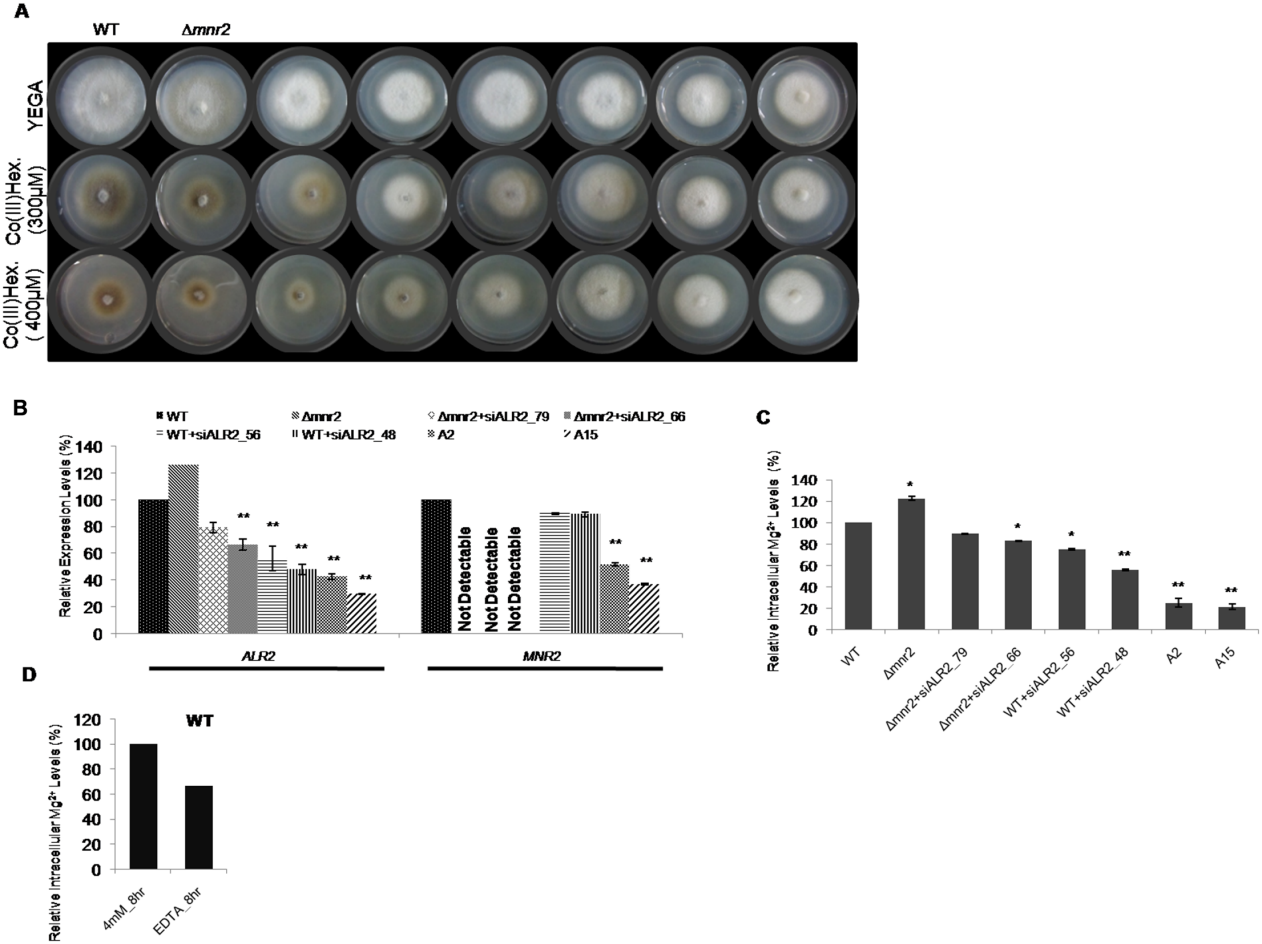
CorA Mg^2+^ transporters affect metal ion composition in *M*. *oryzae*. (A) CorA specific inhibitor (Cobalt (III) hexaammine (Co (III) Hex.) was added to YEG medium at concentrations of 300μM and 400μM. Sensitivity was assessed relative to Wild type (WT) five days post inoculation and growth was measured for WT, *Δmnr2* and knockdown transformants. (B) mRNA levels of *MoALR2* and *MoMNR2* were estimated by qRT-PCR. Transcript levels were normalized to that of WT. (C) Intracellular levels of Mg^2+^ in the knockout and knockdown transformants were estimated by XRF. The values are expressed as percentage values, with 100 corresponding to WT at 4mM Mg^2+^. (D) Intracellular levels of Mg^2+^ in WT were estimated in presence of 4mM extracellular Mg^2+^ and EDTA at 8hrs. The values are expressed as percentage values, with 100 corresponding to 4mM Mg^2+^. ** means P value at <0.0001 and * means significant at P value <0.05. Values are the mean of two independent experiments with each performed in triplicates.

To be sure that reduced transcript levels translated into decline in transporter levels, we used a Co(III) Hexaammine sensitivity test. Unlike WT, growth of CorA knockout/knockdown strains is not sensitive to cation hexaammines, demonstrating that the inhibition is mediated by an interaction between CorA and the hexaammines [25]. We tested the sensitivity of knockdown transformants as compared to WT. Two independent knockdown transformants from each category, namely Alr2 silencing (WT+siALR2), *Δmnr2*+siALR2 and simultaneously silenced for *MoALR2* and *MoMNR2*, which were least sensitive to Co(III)Hex., were selected for further study. Southern blot analysis confirmed single site integration in these knockdown transformants (S2A Fig). The degree of resistance to Co(III)Hex. of the selected transformants, *Δmnr2*+siALR2_79, *Δmnr2*+siALR2_66, WT+siALR2_56, WT+siALR2_48, A2 and A15 (Fig 2A) correlated with the degree of silencing of *MoALR2*. The expression levels (%) of *MoALR2* and *MoMNR2* in the transformants relative to WT were *Δmnr2* (126, 0), *Δmnr2*+siALR2_79 (79, 0), *Δmnr2*+siALR2_66 (66, 0), WT+siALR2_56 (56, 90), WT+siALR2_48 (48, 89), A2 (43, 52) and A15 (30, 37) (Fig 2B). Interestingly, the *Δmnr2* knockout was more sensitive to Co(III)Hex. than wild type, possibly due to up-regulation of *MoALR2* transcripts observed in the three independent *Δmnr2* knockouts. Failure of *Δmnr2* to show Co(III)Hex. resistance, once again suggests its intracellular localization, since cation hexaammines are known to affect only surface transporters, as seen with *MoALR2* knockdowns.

Western blot analysis of selected transformants was done to determine whether protein levels were also reduced in them. The sequence of the CorA domain from MoMnr2 has 50% identity with that of MoAlr2, so the antibodies raised against the CorA domain detected both MoAlr2 (70 kDa) and MoMnr2 (90 kDa). The transformants studied showed lower protein levels of these CorA Mg^2+^ transporters than wild type B157 (S2B Fig).

We next investigated whether a decrease in Mg^2+^ transporter levels affects the intracellular levels of metal ions (mainly Mg^2+^ and Ca^2+^), using X-ray Fluorescence Analysis (XRF) of hyphae obtained under standard growth conditions (medium containing 4mM Mg^2+^). We found a decrease in the intracellular levels of Mg^2+^ in the knockdown transformants (Fig 2C) while intracellular levels of Ca^2+^ did not change significantly (data not shown). Significant decreases in Mg^2+^ levels were seen when the silencing of *MoALR2* was >50%. For instance, XRF analysis of A2 and A15 showed that Mg^2+^ levels were reduced to 25% and 21% of WT levels respectively. Thus we show that CorA Mg^2+^ transporters play a significant role in maintenance of intracellular metal ion composition. Next, to look at the effect of extracellular Mg^2+^ availability, we determined the intracellular Mg^2+^ levels in presence of extracellular EDTA (a Mg^2+^ chelator). The intracellular levels of Mg^2+^ in WT decreased in presence of EDTA (Fig 2D) indicating a need for extracellular Mg^2+^ and its uptake for maintenance of the intracellular ionic milieu.

**Fig 3:**
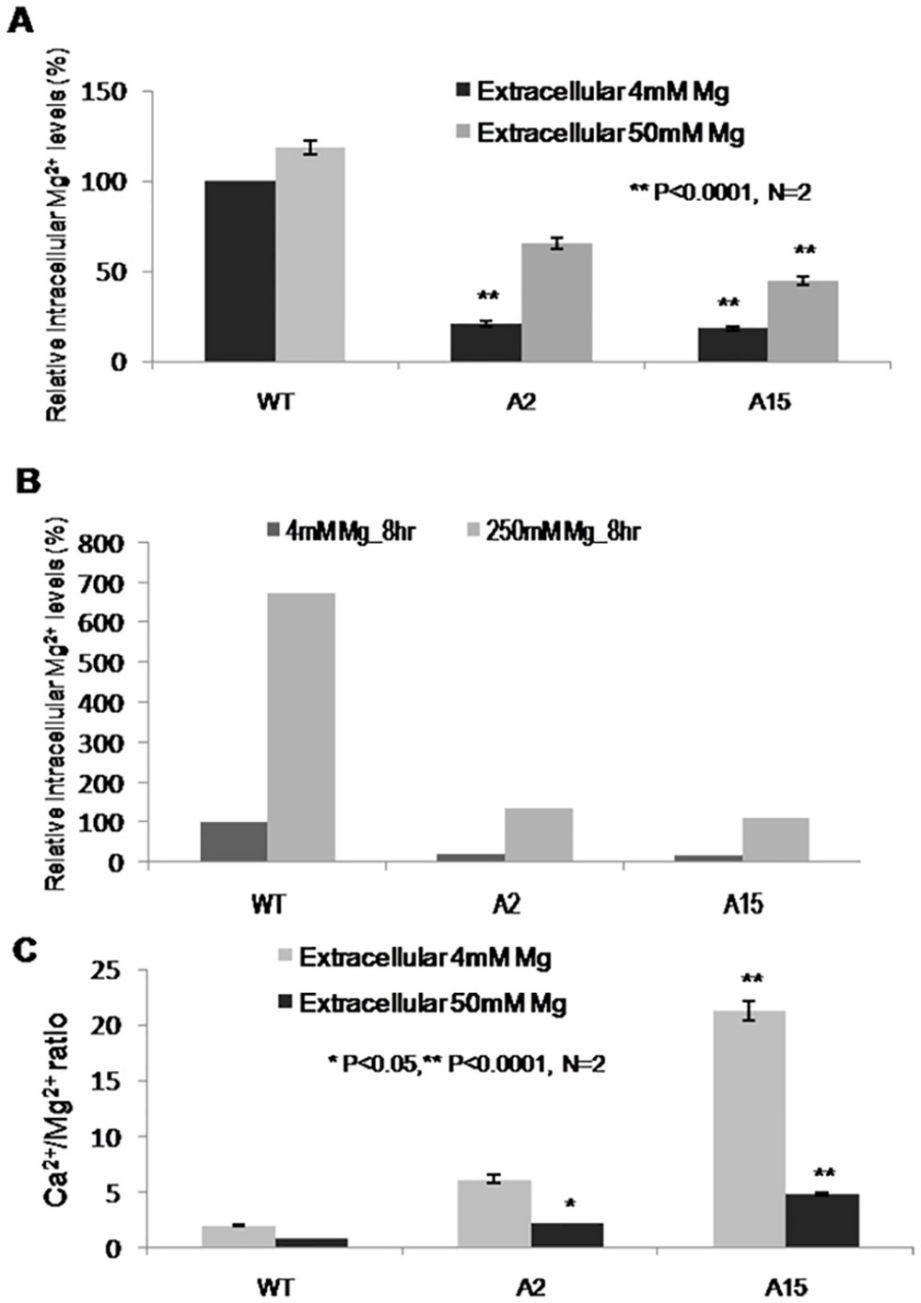
XRF analysis of knockdown transformants. (A), (B) Intracellular levels of Mg^2+^ at 4mM, 50mM and 250mM extracellular Mg^2+^ in WT and the double knockdown transformants, A2 and A15. The values are expressed as percentages, with 100 corresponding to the WT at 4mM Mg^2+^. (C) Ratios of Ca^2+^ to Mg^2+^ at two different concentrations of Mg^2+^ in WT, A2 and A15. Values are the mean of two independent experiments with each performed in triplicates. Error bar denote SD. ** means P value at <0.0001 and * means significant at P value <0.05.

To investigate whether extracellular Mg^2+^ supplementation restores intracellular Mg^2+^, the two knockdown transformants which showed maximum silencing of *MoALR2*, A2 and A15, were grown in presence of higher concentrations of extracellular Mg^2+^ (50mM and 250mM). When supplemented with 50mM Mg^2+^, the intracellular Mg^2+^levels in A2 and A15 increased to 62% and 42% (from 25% and 21%) respectively (Fig 3A) and rose further at 250mM Mg^2+^ (Fig 3B). This could be either due to enhanced uptake by the CorA transporters in the knockdown transformants, or due to non-specific transport at higher levels of Mg^2+^ by other metal ion transporters. In WT, while intracellular Mg^2+^ levels remained unchanged at 50mM extracellular Mg^2+^, there was a drastic increase at 250mM Mg^2+^ (Fig 3A and 3B). The increased Ca^2+^/Mg^2+^ ratio observed in A2 and A15 at 4mM Mg^2+^, also returned to lower levels in presence of 50mM Mg^2+^ (Fig 3C).

### Mg^2+^ dependent expression of *MoALR2* and *MoMNR2*

**Fig 4:**
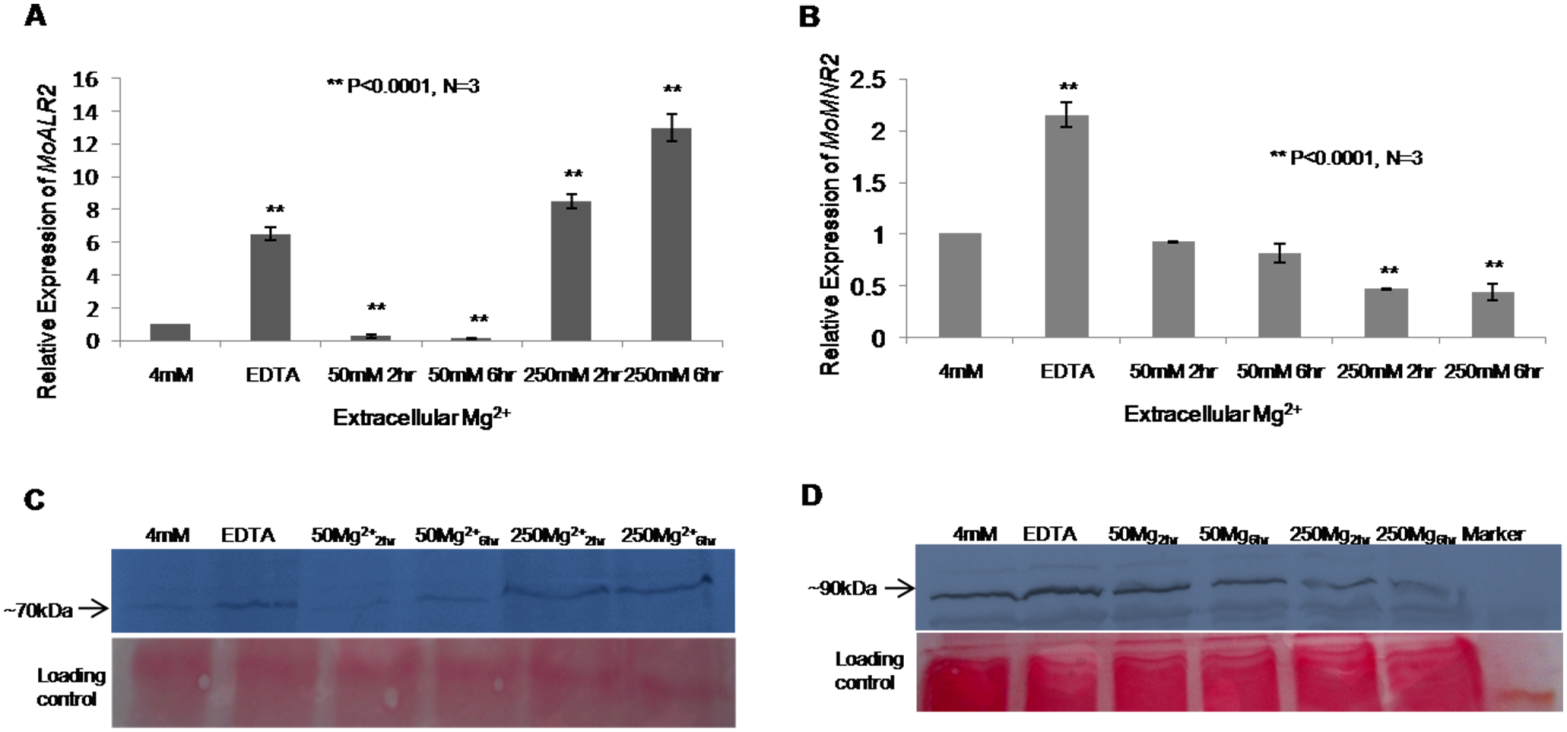
Regulation of *MoALR2* and *MoMNR2*at mRNA and protein level with respect to extracellular Mg^2+^. (A) mRNA levels of *MoALR2* were estimated in WT by qRT-PCR at different concentrations of extracellular Mg^2+^. (B) mRNA levels of *MoMNR2* were estimated by qRT-PCR at different concentrations of extracellular Mg^2+^. Transcript levels were expressed as relative values, with 1 corresponding to levels at 4mM. (C) Western blot analysis of WT for MoAlr2 at different concentrations of extracellular Mg^2+^. (D) Western blot analysis for MoMnr2 at different concentrations of extracellular Mg^2+^. ** means P value at <0.0001 and * means significant at P value <0.05. The experiments were repeated in triplicate, N = 3.

The expression profile of *MoALR2* and *MoMNR2* was studied in WT grown in presence of EDTA, 50mM and 250mM extracellular Mg^2+^ for different lengths of time (2 hours and 6 hours). The addition of EDTA resulted in up-regulation of both *MoALR2* and *MoMNR2*. *MoALR2* showed a biphasic mode of regulation with respect to different concentrations of Mg^2+^; transcript levels decreased at 50mM Mg^2+^ both at 2 hours and 6 hours, while at 250mM Mg^2+^, the transcript level increased both at 2 hours and 6 hours (Fig 4A). The transcript levels of *MoMNR2* decreased with increasing concentrations of Mg^2+^ (Fig 4B). To examine how the levels of MoAlr2 and MoMnr2 proteins change with extracellular Mg^2+^ levels, Western blot analysis was also done. The levels of both proteins increased in presence of EDTA. In the presence of 50mM Mg^2+^ the level of MoAlr2 was comparable to that seen with 4mM Mg^2+^ alone (i.e. no EDTA), both at 2 hours and 6 hours, but increased at 250mM Mg^2+^ both at 2 hours and 6 hours compared to that at 4mm Mg^2+^ (Fig 4C). The level of MoMnr2 protein showed the same trend as seen at transcript level, decreasing with increasing concentration of Mg^2+^ and with increasing time interval (Fig 4D).

### Ca^2+^ dependent expression of *MoALR2* and *MoMNR2*

**Fig 5:**
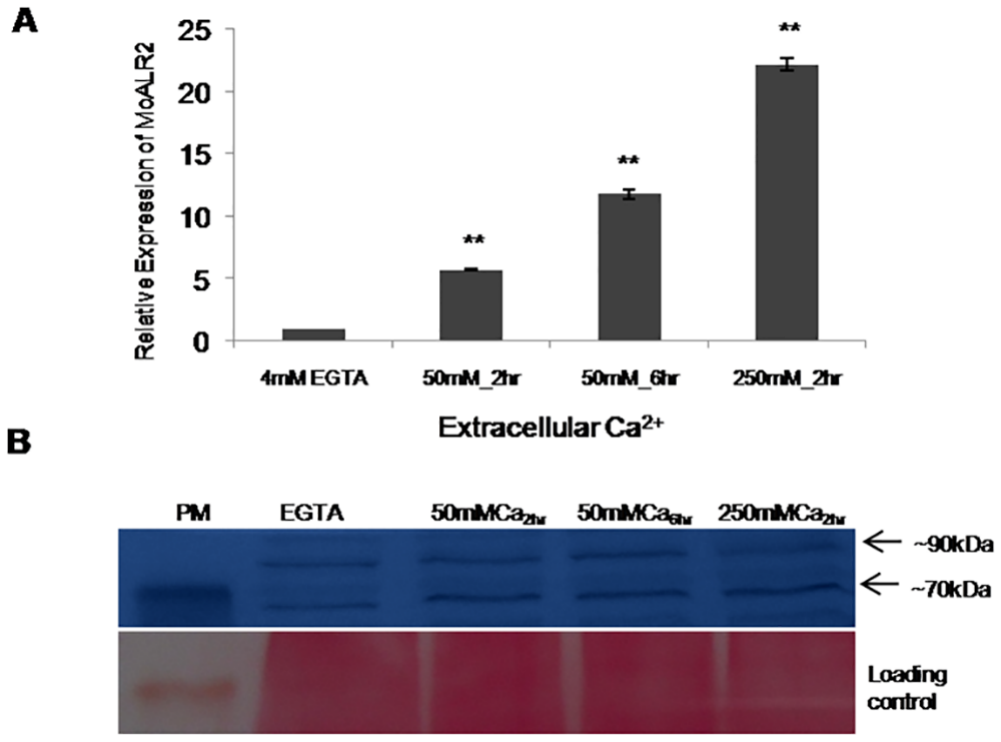
Regulation of *MoALR2* in WT at mRNA and protein level with respect to extracellular Ca^2+^. (A) mRNA levels of *MoALR2* were estimated in WT by qRT-PCR at different concentrations of extracellular Ca^2+^. The transcript levels were expressed as relative values, with 1 corresponding to levels at 4mM. Error bar denote SD. (B) Western blot analysis for MoAlr2 at different concentrations of extracellular Ca^2+^. ** means P value at <0.0001 and * means significant at P value <0.05. The experiments were repeated in triplicate, N = 3.

Calcium (Ca^2+^) is a natural antagonist of Mg^2+^ [23, 9, 14]. To evaluate how expression of the plasma membrane Mg^2+^ transporter *MoALR2* changes with increasing concentration of Ca^2+^, transcript and protein levels were studied in WT. The transcript level of *MoALR2* increased at 50mM and 250mM extracellular Ca^2+^ compared to control (where extracellular Ca^2+^ was chelated with EGTA) (Fig 5A). Protein level of MoAlr2 was higher than in the EGTA-treated control, but MoMnr2 protein level remained constant even at high concentrations of Ca^2+^ (Fig 5B). It is likely that high intracellular concentration of Ca^2+^ induced increased expression of *MoALR2* as part of a feedback mechanism to maintain a favorable Ca^2+^/Mg^2+^ ratio.

### Altered Cation sensitivity in knockdown transformants

Mg^2+^ is the most abundant divalent cation in the cell. A change in Mg^2+^ levels affects metal ion homeostasis and may alter sensitivity to heavy metal ions. We assayed the sensitivity of *Δmnr2* and the knockdown transformants to various cations in comparison to WT. A2 and A15 showed enhanced sensitivity to Aluminium (Al^3+^) (Table 1).

**Table 1:** Altered Cation Sensitivity in the knockdown transformants.

Transformants	Growth diameter (centimeters)							
	YEGA	800μM Al^3+^	750μM Cu^2+^	2mM Fe^3+^	200μM Ni^2+^	150μM Co^2+^	500μM Zn^2+^	3mM Mn^2+^
WT	2.5 ±0.06	2.03 ±0.09	1.97 ±0.04	0.74 ±0.03	0.83 ±0.03	0.37 ±0.04	0.34 ±0.04	1.57 ±0.04
*Δmnr2*	2.47 ±0.03	2.25 ±0.03 ^a^	1.94 ±0.04	0.57 ±0.04^b^	0.54 ±0.04 ^a^	0.36 ±0.03	0.33 ±0.04	0.37 ±0.04 ^a^
*Δmnr2* +siALR2_79	2.3 ±0.07^b^	1.47 ±0.03 ^a^	1.3 ±0.06 ^a^	0.38 ±0.04 ^a^	0.87 ±0.04	0.37 ±0.03	0.33 ±0.04	0.37 ±0.04 ^a^
*Δmnr2* +siALR2_66	2.28 ±0.02^a^	1.43 ±0.03 ^a^	1.23 ±0.03 ^a^	0.34 ±0.04 ^a^	1.02 ±0.05 ^b^	0.34 ±0.04	0.67 ±0.03 ^a^	0.84 ±0.03 ^a^
WT +siALR2_56	2.27 ±0.01 ^a^	1.36 ±0.03 ^a^	1.07 ±0.04 ^a^	0.34 ±0.03 ^a^	1.03 ±0.04 ^b^	0.33 ±0.04	0.66 ±0.02 ^a^	1.27 ±0.04 ^a^
WT +siALR2_48	2.25 ±0.03 ^a^	1.23 ±0.04 ^a^	0.87 ±0.03 ^a^	0.33 ±0.03 ^a^	1.07 ±0.03 ^a^	0.40 ±0.01	0.87 ±0.03 ^a^	1.20 ±0.06^a^
A2	2.08 ±0.05 ^a^	1.02 ±0.04 ^a^	0.9 ±0.01 ^a^	0.30 ±0.01 ^a^	1.04 ±0.04 ^a^	0.87 ±0.04 ^a^	1.63 ±0.04 ^a^	1.80 ±0.02 ^a^
A15	1.95 ±0.03 ^a^	0.74 ±0.03 ^a^	0.73 ±0.04 ^a^	0.29 ±0.01 ^a^	1.27 ±0.04 ^a^	1.03 ±0.04 ^a^	2.03 ±0.03 ^a^	1.97 ±0.03 ^a^

WT, *Δmnr2* and knockdown transformants were inoculated on YEG and YEG supplemented with different cations. The sensitivity to cation was assessed relative to growth of WT. Data are presented as mean±SD from three independent experiments. Two-way ANOVA followed by Fisher’s LSD test was performed at 95% confidence interval.

^a^ means significantly different from WT at P value <0.0001

^b^ means significantly different from WT at P value <0.05.

This is consistent with the observation in *S*. *cerevisiae* that over-expression of the Mg^2+^ transporter provides resistance to Al^3+^, justifying the **ALR** (**Al**uminium **R**esistance) nomenclature. The knockdown transformants were also more sensitive to Copper (Cu^2+^) and Iron (Fe^3+^) (Table 1). Their increased sensitivity towards these cations suggests that the level of Mg^2+^ required to provide resistance against these cations is dependent on *MoALR2* function. Conversely, A2 and A15 were more resistant to Nickel (Ni^2+^), Cobalt (Co^2+^), Zinc (Zn^2+^) and Manganese (Mn^2+^) (Table 1). The *Δmnr2* knockout, on the other hand, showed greater sensitivity to Ni^2+^, Co^2+^, Zn^2+^ and Mn^2+^. These cations have been reported to be transported by CorA transporters [26, 27], so the higher resistance of the knockdown transformants to them is likely to be due to reduced uptake by the lower number of Mg^2+^ transporters. This is also supported by the intracellular levels of Zn^2+^ in A2 and A15, in which Zn^2+^ levels were reduced to 78% and 61% of WT levels (S3A Fig).

### CorA transporters are required for mycelial growth and surface hydrophobicity in *M*. *oryzae*

**Fig 6:**
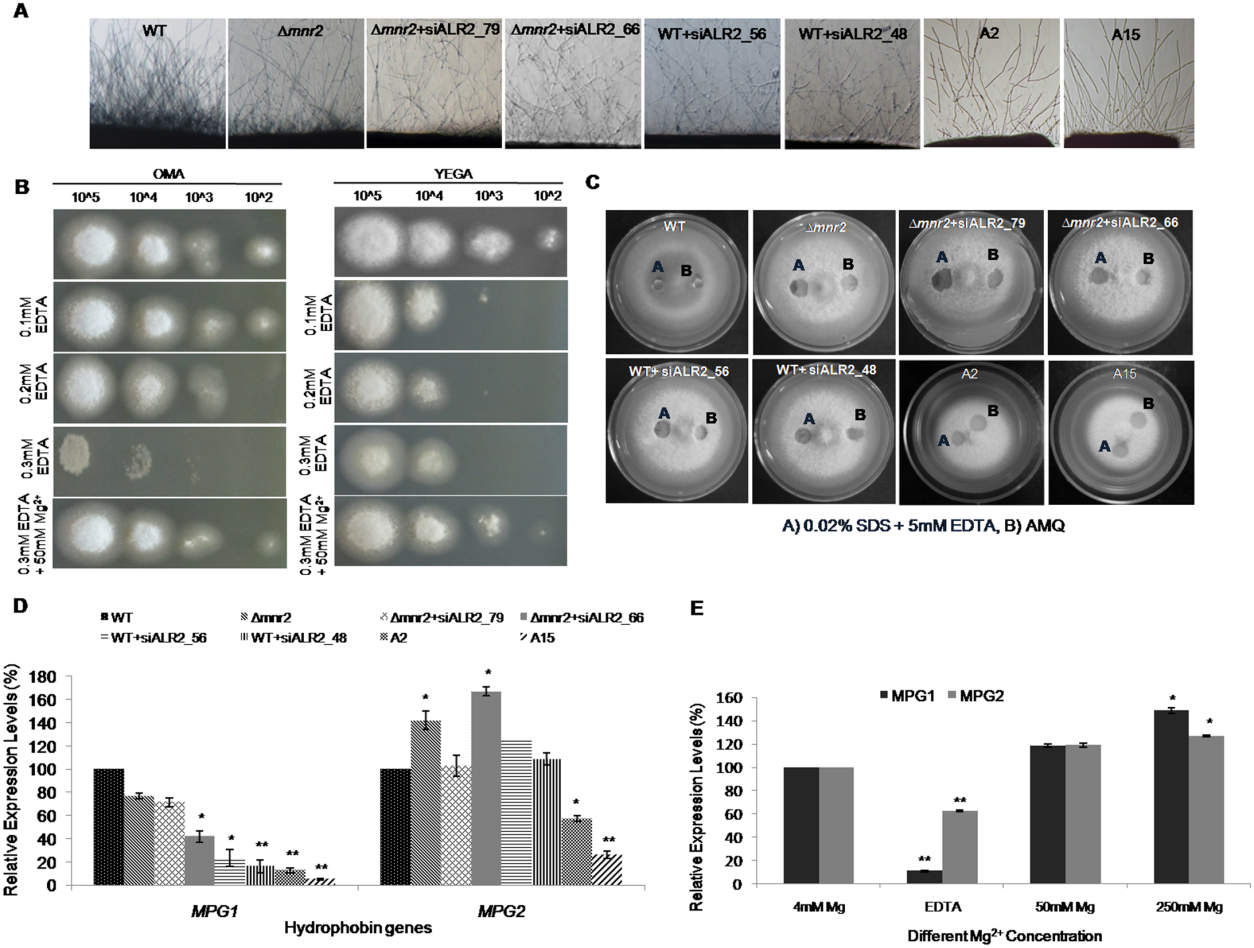
CorA transporters are required for mycelial growth and surface hydrophobicity in *M*. *oryzae*. (A) Microscopic examination of the hyphal growth of WT, *Δmnr2* and knockdown transformants was assessed. Pictures were taken after 2dpi grown on 0.8% agarose. (B) Ability of WT spores to form vegetative hyphal growth following germination was assessed on OMA (left) and YEGA (right) with different concentrations of EDTA. 10μl of spores with increasing dilution was spotted onto the plates. The ability of Mg^2+^ to restore the germination capability of spores was also checked on Mg^2+^ supplemented medium in presence of EDTA. (C) 10 μl of water or detergent solution containing 0.02% SDS+5mM EDTA were placed on the surfaces of the WT, *Δmnr2* and knockdown transformants and photographed after 1 min. (D) mRNA levels of *MoMPG1* and *MoMPG2* were estimated by qRT-PCR in *Δmnr2* and knockdown transformants. All transcript levels were normalized to that of WT. (E) mRNA levels of *MoMPG1* and *MoMPG2* were estimated by qRT-PCR at two different concentrations of extracellular Mg^2+^ in WT. All transcript levels were expressed as relative values, with 1 corresponding to levels at 4mM. Error bar denote SD. ** means P value at <0.0001 and * means significant at P value <0.05. The experiments were repeated in triplicate, N = 3.

We next set out to evaluate the effect of reduced Mg^2+^ transport on development in *M*. *oryzae*. *Δmnr2* showed ~5% reduction in colony diameter as compared to WT and failed to produce melanin in the aerial hyphae (S3B Fig). The *MoALR2* knockdown transformants showed ~6% to 25% reduction in growth on Oat Meal Agar (OMA) medium (S3B Fig) (S3 Table) and this reduction was correlated with the degree of silencing of *MoALR2*. In comparison to WT, *Δmnr2* formed fewer aerial hyphae, while the knockdown transformants showed very sparse aerial hyphae, as seen in A2 and A15 (Fig 6A).

To investigate whether similar growth defects are also observed in situations of low Mg^2+^ availability in *M*. *oryzae*, growth of WT was assayed in Mg^2+^ limiting conditions, using EDTA to lower Mg^2+^ availability. Growth on OMA was severely reduced with increasing concentrations of EDTA, being retarded significantly at 0.5mM EDTA (S4A Fig). On supplementing this medium with 50mM and 250mM extracellular Mg^2+^, growth was restored to normal (S4B Fig). At a concentration of 0.6mM EDTA, there was complete growth inhibition. Under 0.5mM EDTA stress, WT frequently formed sectored colonies with certain sectors showing phenotypic differences (less melanized whitish sectors and melanized grayish sectors). Given the frequency with which such sectors appeared, it is likely that their altered phenotypes were due to epigenetic changes. When the phenotypically different sectors (grown on OMA without EDTA) were grown again in the absence of 0.5mM EDTA stress, these differences (less melanized and whitish) persisted, as observed up to 6 sub-culturings (S4B Fig).

Vegetative hyphal growth from WT spores following germination was checked on OMA and YEGA with increasing concentrations of EDTA. At 0.3mM EDTA growth from spores was severely restricted, while 50mM Mg^2+^ could rescue the growth defect (Fig 6B).

Hydrophobins are surface proteins produced by filamentous fungi that are important for growth of aerial hyphae, hyphal surface hydrophobicity and attachment to solid supports [28–31]. Reduced surface hydrophobicity leads to a “wettable” phenotype where water droplets are not retained as beads on aerial hyphae. Such a wettable phenotype has been observed previously in *M*. *oryzae* in hydrophobin (*MoMHP1*, *MoMPG1*) and phosphodiesterase (*MoPDEH*) mutants [6, 32–34]. *Δmnr2* and *MoALR2* knockdown transformants were tested for their ability to retain drops of water and detergent solution to assess effects of *MoALR2* and *MoMNR2* on surface hydrophobicity. Compared to WT, *Δmnr2* showed a wettable phenotype both with water and detergent solution, but could hold water longer than the *MoALR2* knockdown transformants, which showed an easily wettable phenotype (did not retain solution at all) (Fig 6C). Thus, while *MoMNR2* has some effect on surface hydrophobicity, *MoALR2* appears to be the critical determinant. To investigate whether this wettable phenotype was mediated through hydrophobins, we measured the expression levels of hydrophobins, *MoMPG1* (Mgg_10315) and *MoMPG2* (Mgg_01173), in the *Δmnr2* and knockdown transformants. There was substantial decrease in the expression of *MoMPG1* in the knockdown transformants and in A15 the levels decreased by ~95%. The levels of *MoMPG2* did not change significantly (at P<0.0001) (Fig 6D).

To check whether extracellular Mg^2+^ availability affects expression of *MoMPG1* and *MoMPG2*, their transcript levels in WT were studied at different concentrations of Mg^2+^ and in presence of EDTA. In presence of EDTA, the expression of *MoMPG1* decreased to as little as 10% while *MoMPG2* still showed 60% expression (significant at P<0.0001) (Fig 6E). At 50mM and 250mM Mg^2+^ the expression of *MoMPG1* and *MoMPG2* was similar to that of control at 4mM. Thus, we show for the first time that in *M*. *oryzae*, decrease in Mg^2+^ levels, either by silencing of transporter function (in the knockdown transformants), or by using EDTA (in WT), has a direct effect on the expression of both hydrophobins, especially *MoMPG1*.

**Fig 7:**
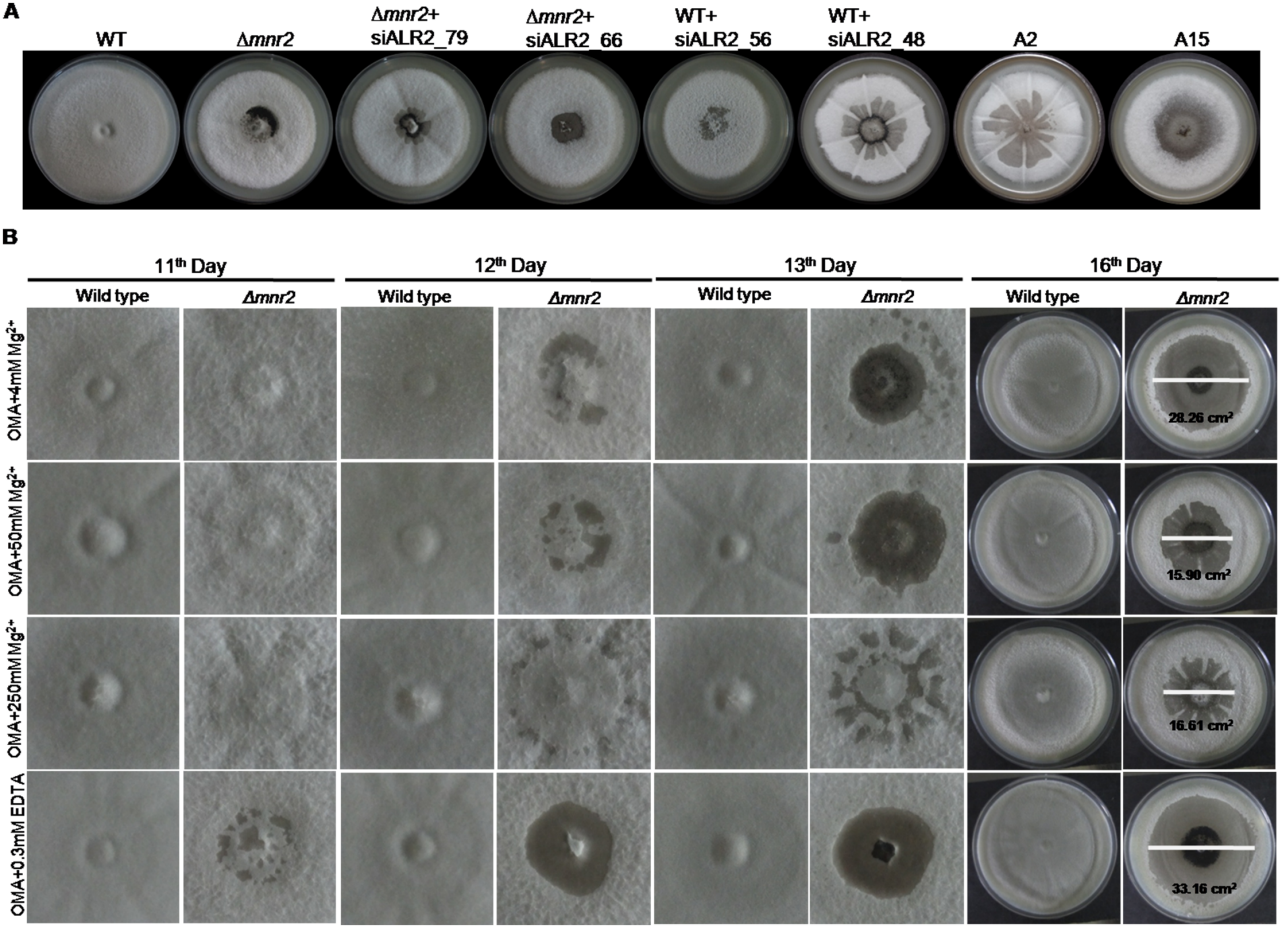
CorA transporters are required for preventing autolysis. (A) WT, *Δmnr2* and knockdown transformants were grown on OMA for 12 days. Early autolysis was monitored compared to WT. (B) WT and *Δmnr2* were grown on OMA supplemented with 4mM, 50mM, 250mM extracellular Mg^2+^ and 0.3mM EDTA. Autolysis was monitored from 10 dpi to 16 dpi and area under autolysis was measured at 16dpi.

Hyphal growth on OMA was observed at regular time intervals post inoculation. As early as 12 days post inoculation (dpi), *Δmnr2* and *MoALR2* knockdown transformants displayed autolysis at the centre of the colony (Fig 7A). WT did not show any such phenotype even up to three weeks. The autolysis was more severe in transformants with *MoALR2* expression below 50%, namely, WT+siALR2_48, A2 and A15 (Fig 7A). We followed *Δmnr2* for a longer time period under different extracellular Mg^2+^ concentrations. Though at 12dpi *Δmnr2* showed autolysis only at the centre, by 16 dpi, autolysis had spread to include a large proportion of the *Δmnr2* colony. 50mM and 250mM extracellular Mg^2+^ supplement delayed the onset of autolysis and as a result the autolysis area observed at 16 dpi was reduced (Fig 7B). On the contrary, EDTA hastened the process with *Δmnr2* displaying the phenotype even at 11 dpi with increasing severity on subsequent days (Fig 7B). In spite of wild type levels of *MoALR2*, *Δmnr2* showed early autolysis compared to WT, suggesting that *MoMNR2* is essential for long term survival of *M*. *oryzae*.

### Magnesium uptake by CorA transporters is essential for progression of the infection cycle in *M*. *oryzae*

**Fig 8:**
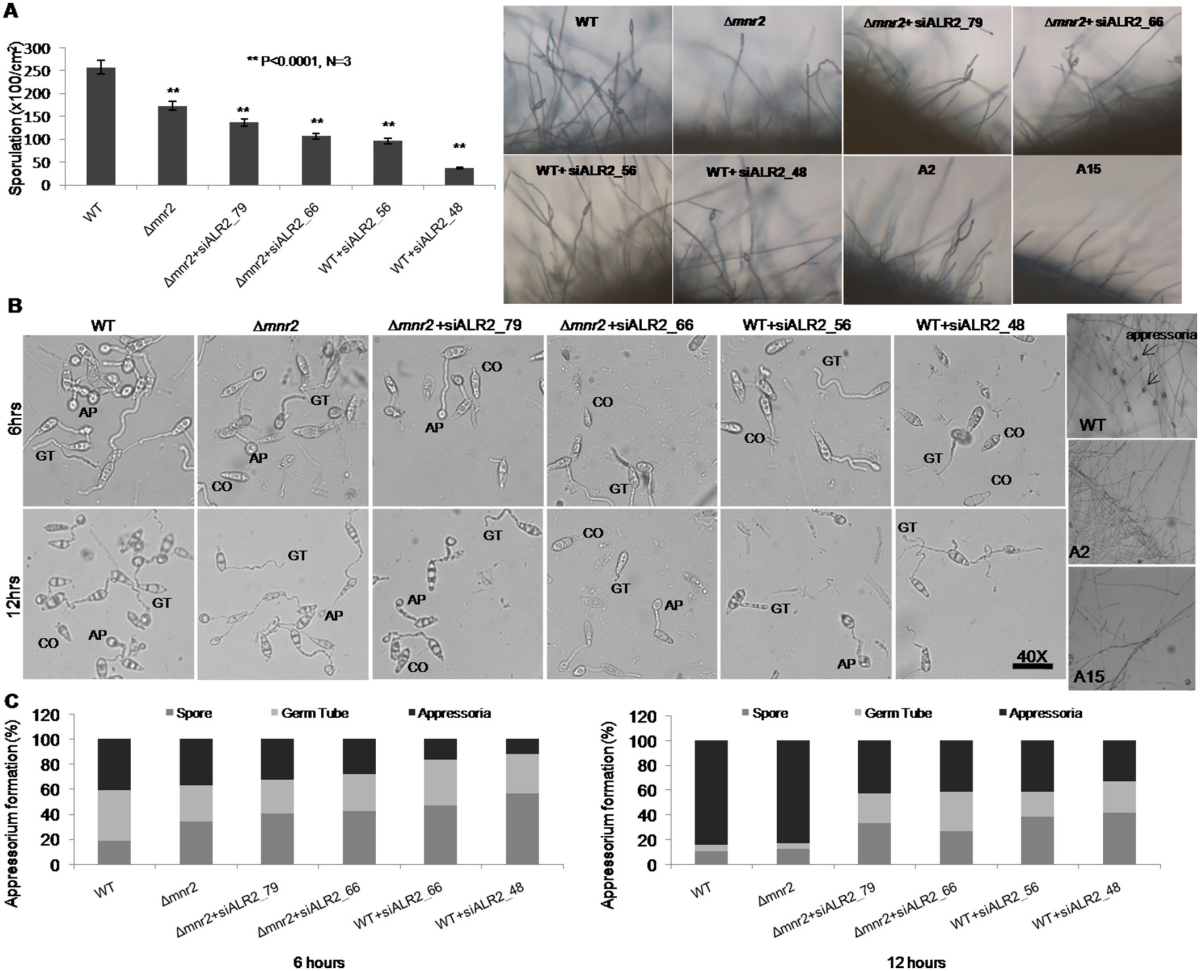
CorA transporters are required for Sporulation and Appressorium formation. (A) Ability of *Δmnr2* and knockdown transformants to sporulate was checked on OMA 8 days post inoculation and quantified. Aerial hyphal and conidial developmentwere also assessed for *Δmnr2* and knockdown transformants at 48 hpi. (B), (C) Appressorial assay for *Δmnr2* and knockdown transformants was performed on hydrophobic gelbond film and the ability to form infection structure was assessed and quantified at 6 hours and 12 hours (CO-Conidium, GT-Germ tube, AP-Appressorium). The values are represented as percentage of spore (ungerminated), germ tube and appressoria formed at the given time interval. Mycelial blocks were placed on hydrophobic surface and incubated upto 72 hours at 28°C for non-sporulating transformants. The experiments were repeated in triplicate, N = 3.

The ability of pathogenic fungi to sporulate is critical to the spread of infection. *Δmnr2* knockout showed a 23% reduction in spore count. In the *MoALR2* knockdowns, sporulation efficiency decreased with a reduction in the expression of *MoALR2*, to as low as 20% in WT+siALR2_48 (Fig 8A). A2 and A15 completely failed to sporulate, suggesting that maintenance of *MoALR2* levels is critical for conidiogenesis.

We studied the ability of WT to sporulate in presence of EDTA. Sporulation increased up to 0.3mM EDTA as compared to control (OMA with no EDTA), and then decreased at higher concentrations (S5A Fig). At 0.5mM EDTA, sporulation was severely decreased. Mg^2+^ supplementation of 250mM was able to rescue this decrease in the sporulation at 0.5mM EDTA, suggesting that adequate Mg^2+^ levels are required for sporulation.

During infection, the spores germinate and differentiate into appressoria. To evaluate the role of *MoALR2* and *MoMNR2* in germination and appressorium formation in WT and knockdown transformants, percentage of spores that germinated and formed appressoria at 6 and 12 hours was determined (Fig 8B and 8C). While in WT 90% of spores had germinated by 12 hours, in knockdown transformants the percentage of ungerminated spores ranged from 33% in *Δmnr2*+siALR2_79 to 41% in WT+siALR2_48. In WT, the percentage of appressoria formed increased from 41% at 6 hours to 85% at 12 hours. In *Δmnr2* the percentage of appressoria formed at 12 hours was 83%, which is comparable to WT. In the *MoALR2* knockdown transformant WT+siALR2_48, the percentage of appressoria formed at 12 hours was only 33%. The percentage of spores that failed to germinate and form appressoria increased with higher level of silencing in the knockdown transformants (Fig 8C). Since A2 and A15 failed to sporulate, mycelial plugs from these transformants were inoculated on hydrophobic surface and incubated under moist conditions for 48 hours (as mycelial tips are also capable of forming appressoria-like structures). The mycelial tips of A2 and A15 failed to develop any appressorium-like structure of the kind seen in the WT (Fig 8B). Thus it is evident that a minimum level of *MoALR2* expression is critical for appressorium formation from germinated spores as well as from hyphae.

**Fig 9:**
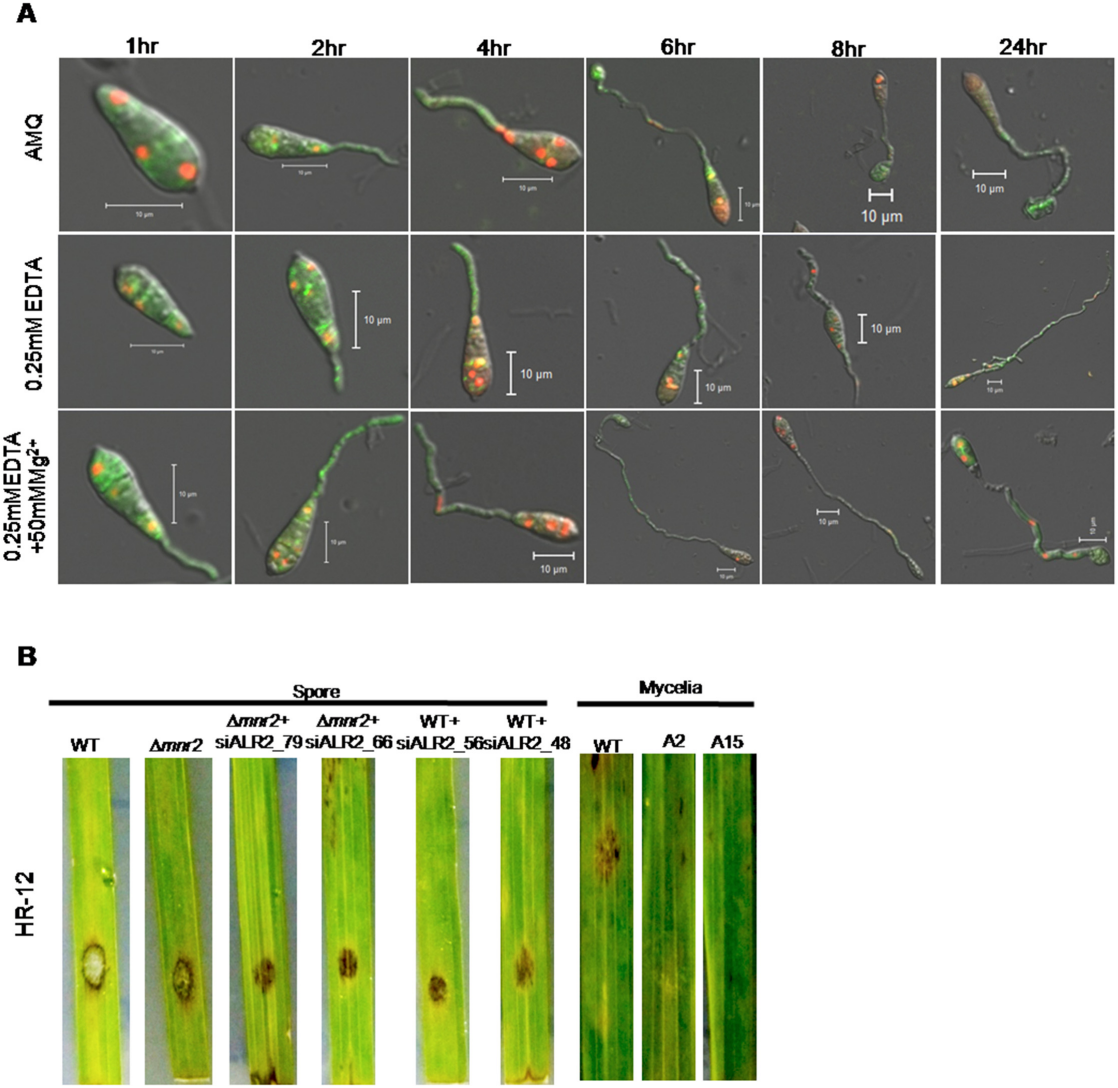
Mg^2+^ uptake by CorA transporters is essential for progression of the infection cycle. (A) Ability to form appressoria in water, 0.25mM EDTA and 0.25mM EDTA+50mM Mg^2+^ was observed at different time intervals in WT tagged with H1:RFP and Tub:GFP. (B) Detached rice leaves of cultivar HR12 were inoculated with spores (1x10^4^/ml) and mycelial plugs. Disease symptoms (lesions) were assessed 4 days post inoculation. The experiments were repeated in triplicate, N = 3.

To test the ability of the conidia to germinate and form appressoria in presence of EDTA and EDTA with 50mM Mg^2+^ supplementation, appressorium formation was studied at different time points in WT (Fig 9A). In the presence of 0.25mM EDTA, even at 24 hours the spores failed to form appressoria (for every time point n>100). Most of the spores (63%) were stalled in the germ tube stage (S5B Fig). Extracellular Mg^2+^ (50mM) was able to rescue the defect in appressorium formation in presence of EDTA in WT. At 24 hours, approximately 53% of the spores had formed appressoria in presence of 50mM Mg^2+^ and 0.25mM EDTA (S5B Fig).

Rice detached leaf infection test showed that the severity of infection decreased in the knockdown transformants (Fig 9B), where small brown lesions were observed as compared to the typical brown bordered gray centered lesions of the WT. The decrease was consistent with the decrease in the levels of *MoALR2*, indicating the importance of *MoALR2* for pathogenicity in *M*. *oryzae*. Thus, extracellular Mg^2+^ availability and transport are critical at all stages of the infection cycle including hyphal growth, conidiation, spore germination, appressorium formation and disease progression in *M*. *oryzae*.

### *MoALR2* affects intracellular cAMP levels in *M*. *oryzae*

**Fig 10:**
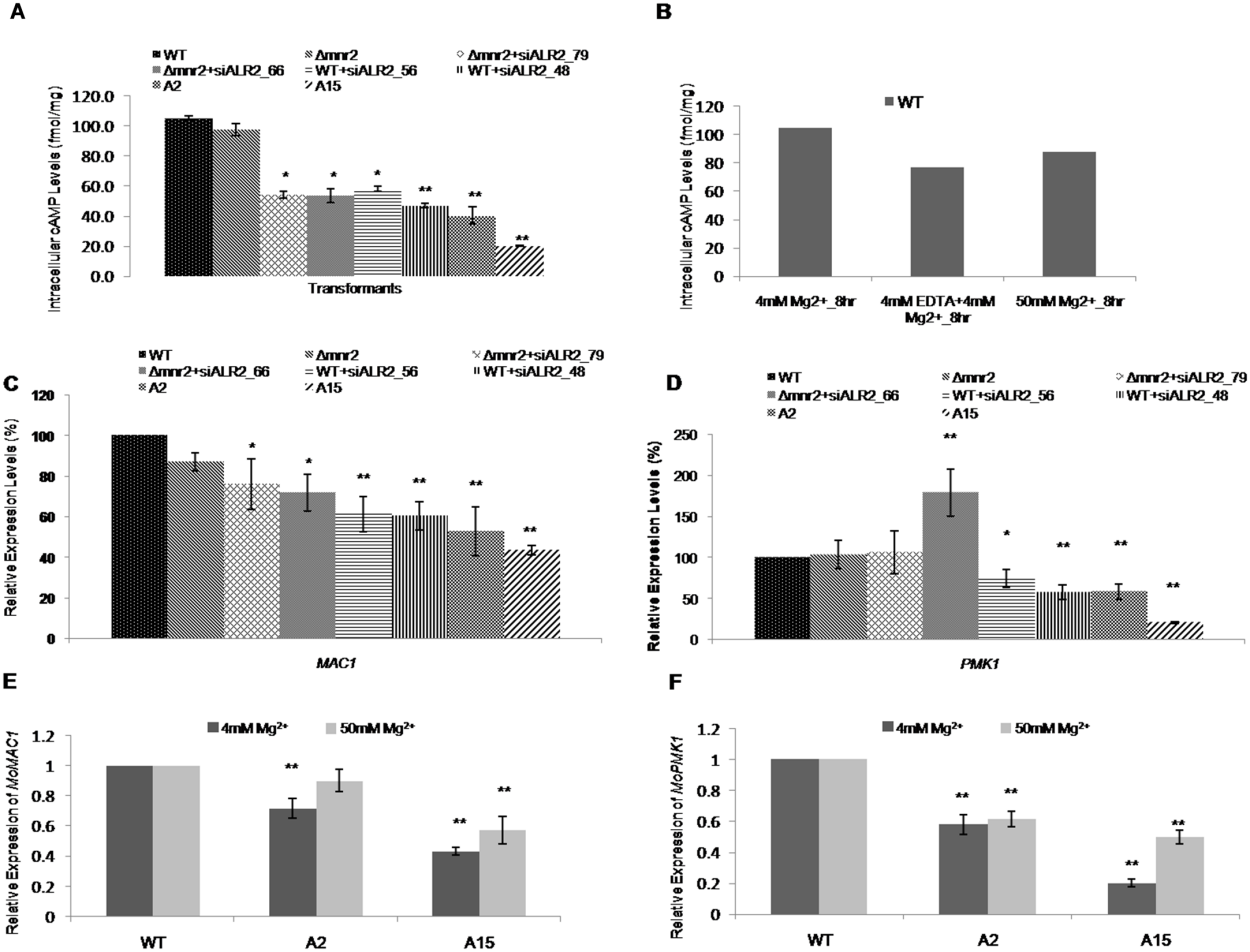
*MoALR2* affects intracellular cAMP levels and cAMP mediated signaling in *M*. *oryzae*. (A) Intracellular cAMP levels were estimated in WT, *Δmnr2* and knockdown transformants. The bar graph represents cAMP levels in fmol mg^-1^. (B) Estimation of intracellular levels of cAMP at 50mM extracellular Mg^2^ in the double knockdown transformants, A2 and A15 in presence of 4mM Mg^2+^, 4mM Mg^2+^ + 4mM EDTA and at 50mM Mg^2+^. The values are expressed in fmol mg^-1^. (C), (D) mRNA levels of *MoMAC1* and *MoPMK1* were estimated by qRT-PCR in WT, *Δmnr2* and knockdown transformants. (E), (F) mRNA levels of *MoMAC1* and *MoPMK1* were estimated by qRT-PCR at two different concentrations of extracellular Mg^2+^ in WT, A2 and A15. The transcript levels were normalized to that of WT. Error bar denote SD. ** means P value at <0.0001 and * means significant at P value <0.05. The experiments were repeated in triplicate, N = 3.

In *M*. *oryzae*, cAMP mediated signaling through *MoPMK1* (Mgg_09565) is crucial for conidiation and appressorium initiation [7, 35]. cAMP is synthesized by Mg^2+^ dependent adenylate cyclase [14, 36]. In view of the low Mg^2+^ levels in the knockdown transformants, we looked for changes in intracellular cAMP levels. The levels of cAMP in *Δmnr2* and the knockdown transformants ranged from 97to 20 fmol mg^-1^ as compared to 105 fmol mg^-1^ in WT (Fig 10A). The cAMP levels correlated with the transcript levels of *MoALR2* and intracellular Mg^2+^ levels in the transformants. In WT, cAMP levels decreased in presence of EDTA (Fig 10B) and were consistent with the intracellular levels of Mg^2+^ in presence of EDTA described earlier (Fig 2D). cAMP levels were restored at 50mM Mg^2+^ at 8 hours in WT (Fig 10B). In presence of EDTA, cAMP levels in A2 and A15 did not correlate to intracellular Mg^2+^ levels (data not shown).

We studied the expression of the adenylate cyclase gene, *MoMAC1* (Mgg_09898) and of *MoPMK1* (encoding MAPK) in the knockdown transformants and found significant decrease in expression, with the transcript levels decreasing to 43% and 20% respectively in A15 (Fig 10C and 10D). To study whether extracellular Mg^2+^ affects expression of *MoMAC1* and *MoPMK1*, we looked at their transcript levels in A2 and A15 with 50mM Mg^2+^ supplementation, and found that expression was restored to WT levels (Fig 10E and 10F). Since the knockdown transformants of *MoALR2* showed decreased ability to conidiate and form appressoria, the low level of intracellular cAMP is likely to be one of the factors contributing to the defect seen in appressorium formation in knockdown transformants.

### *MoALR2* knockdown transformants show altered cell wall integrity

Mg^2+^ is vital for the integrity of the cell wall and cell membrane [37, 9–12]. We asked whether decreased Mg^2+^ levels lead to changes in cell wall integrity (CWI). Growth was measured on media containing the cell wall stressors Congo Red (CR) [38] and Caffeine [39–41]. *Δmnr2* transformants were resistant to 1.5 and 2 mg ml^-1^ CR, similar to WT. However the knockdown transformants, both in WT and *Δmnr2* backgrounds, were more sensitive to CR, with A15 showing the maximum sensitivity (Table 2).

**Table 2:** Cell Wall Integrity assay.

Transformants	Growth diameter (centimeters)				
	YEGA	1.5 mg/ml Congo Red	2.0 mg/ml Congo Red	2.5mM Caffeine	3mM Caffeine
WT	2.5 ±0.063	2.26 ±0.020	1.25 ±0.029	1.74 ±0.006	1.40 ±0.013
*Δmnr2*	2.47 ±0.032	1.63 ±0.016 ^a^	1.20 ±0.010	1.57 ±0.016 ^a^	1.34 ±0.013
*Δmnr2*+siALR2_79	2.3 ±0.073^a^	1.69 ±0.020 ^a^	1.19 ±0.011	1.21 ±0.010 ^a^	1.06 ±0.020 ^a^
*Δmnr2*+siALR2_66	2.28 ±0.018 ^a^	1.57 ±0.016 ^a^	1.19 ±0.010 ^b^	1.03 ±0.013 ^a^	0.87 ±0.017 ^a^
WT+siALR2_56	2.27 ±0.012 ^a^	1.57 ±0.019 ^a^	1.17 ±0.013^b^	1.11 ±0.006 ^a^	1.03 ±0.013 ^a^
WT+siALR2_48	2.25 ±0.032 ^a^	1.22 ±0.017 ^a^	1.03 ±0.019 ^a^	1.01 ±0.006 ^a^	0.97 ±0.016 ^a^
A2	2.08 ±0.048 ^a^	1.22 ±0.010 ^a^	1.02 ±0.018 ^a^	0.71 ±0.020 ^a^	0.69 ±0.005 ^a^
A15	1.95 ±0.032 ^a^	1.02 ±0.006 ^a^	0.87 ±0.006 ^a^	0.51 ±0.023 ^a^	0.35 ±0.003 ^a^

2X2 mm mycelial plugs of WT, *Δmnr2* and knockdown transformants were inoculated on YEG and YEG supplemented with Congo Red and Caffeine. Growth was assessed 5 days post inoculation. Data are presented as mean±SD from three independent experiments. Two-way ANOVA followed by Fisher’s LSD test was performed at 95% confidence interval.

^a^ means significantly different from WT at P value <0.0001

^b^ means significantly different from WT at P value <0.05.

The degree of sensitivity towards cell wall stressors increased in proportion to silencing of *MoALR2*. Mg^2+^ supplementation could restore the normal (lower) sensitivity to cell wall stressors (S6 Fig). These results indicate that maintenance of Mg^2+^ levels dependent on *MoALR2* is critical for the integrity of the cell wall.

**Fig 11:**
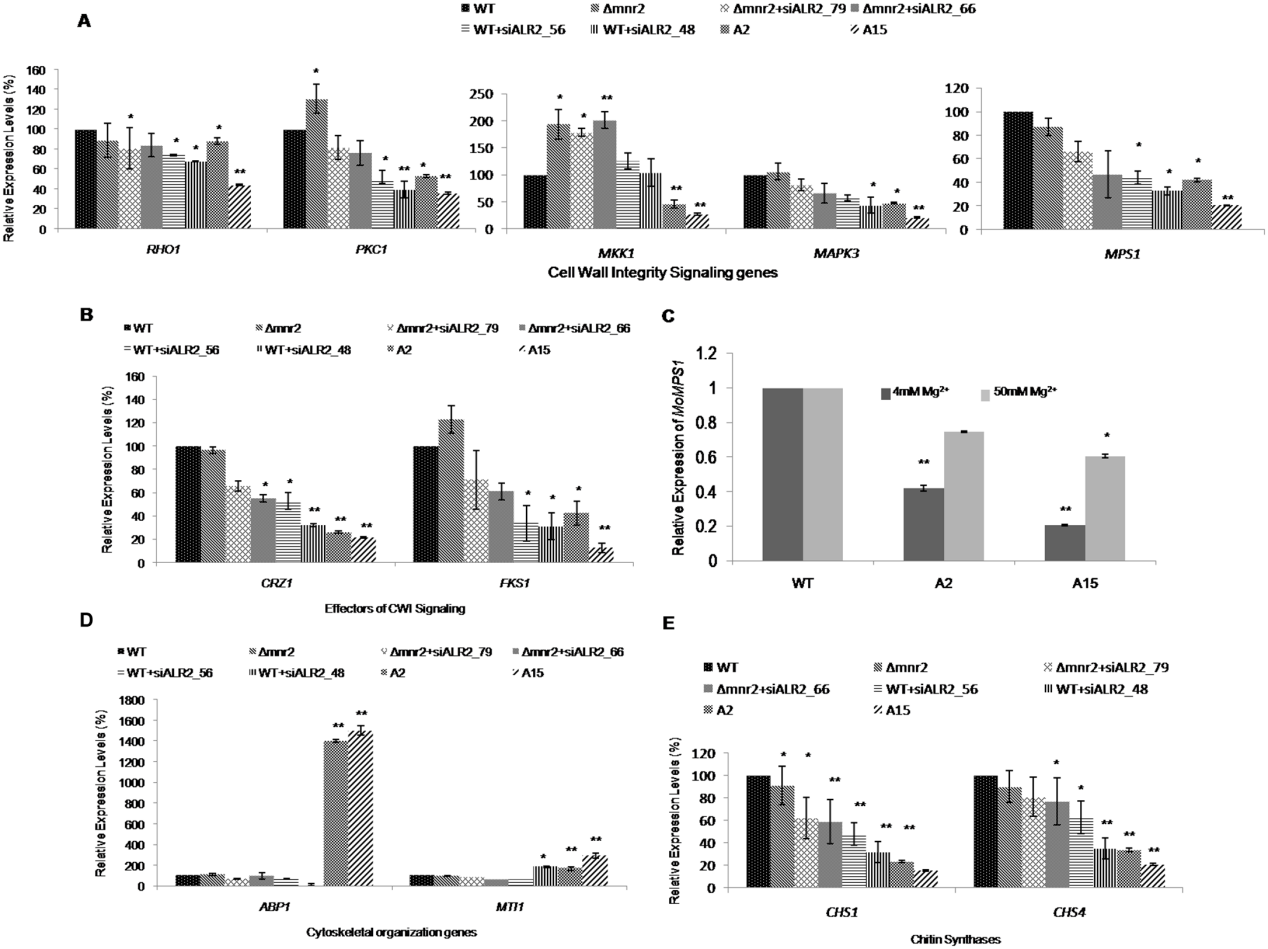
Expression analysis of genes involved in the CWI Pathway in knockout and knockdown transformants. (A) mRNA levels of *MoGTBP1*, *MoPKC1*, *MoMAPK3*, *MoMKK1* and *MoMPS1* were estimated by qRT-PCR. (B) mRNA levels of *MoCRZ1* and *MoFKS1* were estimated by qRT-PCR. (C) mRNA levels of *MoMPS1* were estimated by qRT-PCR at two different concentrations of extracellular Mg^2+^ in WT, A2 and A15. (D) mRNA levels of *MoABP1* and *MoMTI1* were estimated by qRT-PCR. (E) mRNA levels of *MoCHS1* and *MoCHS4* were estimated by qRT-PCR. All transcript levels were normalized to that of WT. Error bar denote SD. ** means P value at <0.0001 and * means significant at P value <0.05. The experiments were repeated in triplicate, N = 3.

To understand the cell wall defects in the knockdown transformants better, we looked at expression of cell wall maintenance related genes. We found decreased expression of two chitin synthase genes, *MoCHS1* (Mgg_01802) and *MoCHS4* (Mgg_09962), in the knockdown transformants (Fig 11E).

We studied the expression of genes involved in the CWI pathway in *Δmnr2* and knockdown transformants. In simultaneously silenced transformants (for *MoALR2* and *MoMNR2*), A2 and A15, there was significant down-regulation of genes involved in the Pkc1 activated mitogen activated protein (MAP) kinase cascade including *MoPKC1*, *MoMKK1*,*MoMAPK3* and *MoMPS1* (at P<0.05) (Fig 11A) only for*MoGTBP* (Mgg_07176) (*Rho1*), did expression not change significantly (at P<0.0001). In contrast *Δmnr2* showed increased expression of *MoPKC1* and *MoMKK1*. Increased expression of *MoMKK1* was also observed in *Δmnr2*+siALR2_79 and *Δmnr2*+siALR2_66 (*MoALR2* silencing in *Δmnr2* background), while no significant changes were observed in WT+siALR2_56 and WT+siALR2_48 (*MoALR2* silencing in WT background).

The expression of downstream effectors of CWI signaling, *MoFKS1* (Mgg_00865) and *MoCRZ1* (Mgg_05133) was also significantly decreased in the knockdown transformants (Fig 11B). The expression of *MoMPS1* in A2 and A15 was restored to WT levels when supplemented with 50mM Mg^2+^ (Fig 11C), indicating that Mg^2+^ levels affect the expression of *MoMPS1*. The decreased expression of CWI signaling genes in the knockdown transformants explains the sensitivity towards cell wall stress and demonstrates the role of *MoALR2* in maintenance of Mg^2+^ levels critical for cell wall integrity.

Actin cytoskeletal reorganisation is an important aspect of the compensatory response to cell wall defects [37]. We analyzed the expression of the genes *MoABP1* (Mgg_06358) and *MoMTI1* (Mgg_04116), in *Δmnr2* and the knockdown transformants by qRT-PCR. There was significantly increased expression of both *MoABP1* and *MoMTI1* in A2 and A15 (at P<0.05) (Fig 11D). The expression of *MoABP1* was 15 fold higher, implying that MoAbp1 might play a role in stabilizing the cytoskeleton and membranes to compensate for the lack of Mg^2+^, which is known to be crucial for maintenance of cell shape and membrane integrity.

## Discussion

Magnesium is an essential mineral nutrient with roles in stability of DNA structure, cell membrane maintenance, activity of ATP, and as a cofactor of several enzymes. In spite of the importance of Mg^2+^ in cellular physiology, there is little information about the transport, regulation and storage of Mg^2+^ in fungi. In this study, we show that the CorA Mg^2+^ transporters MoAlr2 and MoMnr2 play important roles in development and virulence of the model fungal pathogen, *Magnaporthe oryzae*. Our results, supported by the phenotypic defects seen in the knockout and knockdown transformants—*Δmnr2*, WT+siALR2, *Δmnr2*+siALR2 and transformants in which both genes were simultaneously knocked down (A2 and A15)—show that CorA Mg^2+^ transporters are intimately involved at all stages of the infection cycle of *M*. *oryzae*. Chemical inhibition of Mg^2+^ transport by the CorA specific inhibitor Co(III)Hex. in wild type also produced growth defects. We also used the Mg^2+^ chelator EDTA to deplete Mg^2+^ to study the effect of extracellular magnesium availability in the wild type strain B157. Higher levels of EDTA completely abolish growth in WT, while lower levels inhibit spore germination, and an even lower concentration of EDTA is sufficient to inhibit the process of appressorium formation. A corresponding gradation of phenotypes is also observed in the different knockdown transformants, where effects of low silencing are seen on appressorium development. With stronger silencing spore germination too is affected, and at the highest level of silencing we see drastic effects even on hyphal growth, suggesting that during the course of the infection cycle from vegetative hyphal growth to sporulation to appressorium formation, the requirement for Mg^2+^ transport into the cell goes up. Recently, it has been shown that *MoALR2* is down-regulated during *in planta* growth in barley and rice at 72 hours post inoculation (hpi) [42].

The severity of growth defects of transformants was further intensified in low Mg^2+^ conditions; for instance, *Δmnr2* knockout showed less growth than WT in presence of EDTA (S7 Fig). This high Mg^2+^ requirement could be for processes like cell wall remodeling, cell division and maintenance of surface hydrophobicity that form a critical part of the differentiation process. Spore germination and appressorium formation, where we have shown a requirement for MoAlr2, are part of the early events of infection on the plant host and are completed well before 72 hpi. Due to failure to obtain a true knockout for *MoALR2* in spite of the use of diverse approaches, and from the drastic defects observed in the knockdown transformants, we conclude that *MoALR2* is likely essential for viability of the rice blast fungus. Further, we did not obtain any knockdown transformants with less than 30% expression of *MoALR2*, indicating that a critical minimum level of *MoALR2* expression is essential for viability (although it cannot be ruled out that our silencing procedure is unable to achieve a higher level of silencing). Previous large scale random mutagenesis screens for pathogenicity have also not uncovered any mutants of this gene. In all, our results show that the knockdown transformants have defects in hyphal growth, conidiation, spore germination, appressorium formation and infection. Given this requirement for Mg^2+^ transport at all stages of the *M*. *oryzae* infection cycle, CorA transporters may be good targets for the development of antifungal agents. Ion channel blockers, agents that sequester Mg^2+^ from the fungal environment and rice lines expressing RNAi targeting Mg^2+^ transporter could prove fatal to fungal proliferation.

Element analysis in the knockdown transformants showed that decreased levels of MoAlr2 transporter led to lowered intracellular levels of Mg^2+^ (Fig 2C). The knockdown transformant A15, which showed maximum silencing of *MoALR2* and *MoMNR2*, also had the lowest intracellular levels of Mg^2+^. Complementation studies in *S*. *cerevisiae* also suggest that *MoALR2* and *MoMNR2* have a definite role in Mg^2+^ transport. We used the 489–812 amino acid region encompassing the CorA transmembrane helices to carry out complementation and have shown that these 324 amino acids at the C terminus of MoMnr2 are sufficient for the function of Mg^2+^ transport (Fig 1D). Earlier mutagenesis experiments in *ScAlr1* showed similar effects where the 239 amino acids at the N-terminal and 53 amino acids at the C-terminal are not essential for Mg^2+^ uptake [43].

Regulation of Mg^2+^ involves localization, compartmentalization, and sequestration [44]. Higher expression of *MoALR2* was observed in *Δmnr2* transformants. The maximum sensitivity of *Δmnr2* to Co(III)Hex. and higher intracellular levels of Mg^2+^ in *Δmnr2* correlated with the increased levels of *MoALR2*. We suggest that absence of an organellar transporter MoMnr2 in the *Δmnr2* knockout transformant may lead to up-regulation of expression of *MoALR2* encoding the plasma membrane transporter. The external environment of the fungus is dynamic with respect to the levels of divalent cations like Mg^2+^ and Ca^2+^. In *S*. *cerevisiae*, under Mg^2+^ limiting conditions, vacuolar Mg^2+^ contributes towards the maintenance of cytosolic Mg^2+^ levels through the activity of *MNR2* [45]. We show that CorA transporters are regulated in response to changes in the extracellular ionic milieu. *MoALR2* and *MoMNR2* are induced by the depletion of extracellular Mg^2+^, while their levels decreased at higher concentrations of Mg^2+^. *MoMNR2* may be down-regulated to reduce the efflux of Mg^2+^ from organellar stores, thereby preventing toxicity due to increased cytoplasmic levels of Mg^2+^. We found that with increasing concentrations of Ca^2+^, both the transcript and protein levels of *MoALR2* increased. The severity of panicle blast has previously been shown to be positively correlated with Mg^2+^ levels and negatively affected by Ca^2+^ concentration in the plant tissue [46]. Determination of the ionic composition of the leaf and transporter levels in the fungus during invasion and proliferation will shed further light on the significance of Mg^2+^ uptake by CorA transporters at the site of infection.

The *MPG1* hydrophobin gene plays a key role in the development of *M*. *oryzae* and its expression is regulated in response to diverse morphogenetic and environmental signals. The Mpg1 protein at the cell surface perceives stimuli such as surface hydrophobicity and conveys the signal through G protein coupled receptors to activate adenylate cyclase, which in turn activates Pka and Pmk1 dependent MAP kinase, vital for appressorium development and maturation [35]. While MoPmk1 acts downstream of MoMpg1, it is also known in turn to regulate the expression of the *MoMPG1* gene [47]. MoMpg1 is essential for conidiogenesis and appressorium formation and it has been proposed that MoMpg1 may exert positive feedback on its own expression through the cAMP response pathway [47]. *Δmnr2* knockout and *MoALR2* knockdown transformants showed fewer aerial hyphae, surface hydrophobicity defects and a wettable phenotype. Decrease in hydrophobicity in presence of low levels of Mg^2+^ has been previously observed in *S*. *cerevisiae* [48]. Our knockdown transformants also showed lower expression of hydrophobin genes (Fig 7D). We demonstrate that a reduction in Mg^2+^ levels, either by knockdown of transporter function or by using EDTA in the WT, has an effect on the expression of the hydrophobin gene *MoMPG1*. *Δmnr2* showed an early autolysis phenotype. The autolysis occurred even earlier in the knockdown transformants both in WT and *Δmnr2* background and the degree of autolysis correlated with the degree of knockdown of *MoALR2*.

We also found that the intracellular levels of cAMP are lower in the *MoALR2* knockdown transformants. cAMP is an important secondary messenger in the cell and its levels regulate appressorium formation in *M*. *oryzae* [7, 35]. Decrease in intracellular cAMP reduces the flux through the Pmk1 pathway and in turn reduces the expression of *MoMPG1*, thus possibly contributing to the defects in conidiation, appressorium formation and infection seen in*MoALR2* knockdown transformants.

We found that silencing of the Mg^2+^ transporters led to a loss of cell wall integrity, indicating that Mg^2+^ transport is vital for cell wall structure. The cell wall integrity (CWI) signaling cascade has been studied extensively in *S*. *cerevisiae* and involves a Rho1 ‘master regulator’ which activates Pkc1 activated mitogen activated protein (MAP) kinase cascade involving effectors like Pkc1, Bck1, Mkk 1/2 and Mpk1. These effectors regulate a diverse set of processes including β-glucan synthesis at the site of cell wall remodeling, gene expression related to cell wall biogenesis, organization of the actin cytoskeleton, and secretory vesicle targeting to growth sites [49]. We hypothesized that a decrease in Mg^2+^ levels in knockdown transformants affects cell wall structure by alteration of the CWI signaling. Consistent with this, we found a decrease in expression of genes involved in CWI signaling in the knockdown transformants. CWI signaling is important for invasive growth, conidiation and plant penetration in *M*. *oryzae* [50]. The decreased expression of hydrophobins, *PMK1* and genes encoding members of the MoMps1-dependent CWI pathway, and low levels of cAMP in *MoALR2* knockdown transformants are among the major factors contributing to their decreased ability to conidiate, form appressoria and cause infection.

We show that *MoALR2* regulates intracellular Mg^2+^ concentration and modulates key signaling pathways necessary for development and pathogenicity in *M*. *oryzae*. Decrease in the expression of the CorA Mg^2+^ transporter *MoALR2* leads to defects in growth, conidiation and appressorium formation, which are critical features for successful establishment of the pathogen within the host. Overall, we show that the CorA transporter MoAlr2 is the dominant factor for maintenance of Mg^2+^ homeostasis during growth and differentiation in *M*. *oryzae*. Knockdown of CorA Mg^2+^ transporters below a critical level makes the pathogen lose its virulence and hence these transporters are potential targets for anti-fungals. In future, the role of CorA transporters in sub-cellular Mg^2+^ distribution and dynamics of cations between the organelles and the cytoplasm needs to be addressed in greater detail.

## Materials and Methods

### Fungal strain and culture conditions

*Magnaporthe oryzae* B157 strain (MTCC accession number 12236), belonging to the international race IC9 was previously isolated in our laboratory from infected rice leaves [51]. The *Δku80* mutant used in the present study was generated by replacing *MoKU80* ORF with Zeocin selection marker in wild type B157 strain (WT) in our laboratory. The fungus was grown and maintained on YEG medium (Glucose1 g, yeast extract 0.2 g, H_2_O to 100 ml) or Oatmeal agar (Hi-Media, Mumbai, India).

### Complementation of a *S*. *cerevisiae Δalr1Δalr2* mutant

*S*. *cerevisiae Δalr1Δalr2* mutant (CM66) having the genotype *Mata alr1*::*HIS3*, *alr2*::*TRP1*, *his3-200*, *ura3-52*, *leu2-1*, *lys2-202*, *trp1-63* and the strain from which it was derived (CM52) having the genotype *Mata his3-200*, *ura3-52*, *leu2-1*, *lys2-202*, *trp1-63* was used for functional complementation studies. The full length gene of *MoALR2* (1.9kb) was amplified from genomic DNA and cloned at *Pvu*II site in the yeast episomal vector pYES2 (Invitrogen, California, USA) to generate pYES2-*MoALR2*. The full length gene of *MoMNR2* (2.5kb) was amplified from genomic DNA and cloned first at *EcoR*V site in pBluescript KS (+). The full length gene was moved into pYES2 vector at *Hind*III and *BamH*I site under Gal1 promoter to give pYES2-*MoMNR2*. For *MoMNR2*_489-812_ cloning, the CorA domain of *MoMNR2* was PCR amplified from genomic DNA and cloned first in pBluescript KS (+) at *EcoR*V site. The CorA domain of *MoMNR2* was moved into pYES2 vector at *Xho*I and *BamH*I site under Gal1 promoter to give pYES2-*MoMNR2*_489-812_. This construct was used to transform the *S*. *cerevisiae Δalr1Δalr2* double mutant (CM66). Colonies were selected on SD medium lacking uracil and having lysine, leucine, 2% Galactose and 500mM MgSO_4_. Transformed colonies obtained were grown in SD media (lysine + leucine + 2% Galactose + 500mM MgSO_4_) till saturation and cells were spotted on SD media having leucine, lysine, 2% Galactose and 4mM MgSO_4_/500mM MgSO_4_. The growth of colonies was seen 4 days post inoculation at 28°C. All primers used in the study are listed in S4 Table.

### Raising of antibodies againstMoAlr2 and MoMnr2

The CorA domains of MoAlr2 and MoMnr2 have 50% identity at the protein level and the size of the proteins is 70kDa and 90kDa respectively. The CorA domain from *MoMNR2* was amplified (1kb) from genomic DNA as it has no intron. The amplified product was cloned in pBluescript KS (+) vector at *EcoR*V site and then was ligated in bacterial expression vector pET30a (+) vector at *Nde*I and *Kpn*I site translationally in frame with a (His)_6_ tag at the C terminus. *E*. *coli* BL21 DE3 cells were transformed with the protein expression construct. The transformed colonies were grown in Luria Bertani (LB) medium O/N. 1% inoculum was used the next day and the culture was grown to an O.D. (λ_600_) of 0.4 to 0.6 O.D.1mM IPTG was used for induction of the protein for another 4 hours. The induced protein having Polyhistidine at the carboxyl terminus was purified using Ni-NTA affinity chromatography (Novagen, Darmstadt, Germany) according to manufacturer’s protocol. The purified protein was used to raise polyclonal antibodies in New Zealand White Rabbit. Antibody titer was estimated by indirect-enzyme linked immunosorbent analysis (ELISA) using HRP conjugated anti-rabbit IgG (Bangalore Genie, Bangalore, India) as the secondary antibody.

### Indirect immunolocalization of MoAlr2 and MoMnr2 in *M*. *oryzae*

The wild type B157 spores were fixed with 10% formaldehyde, 5% acetic acid, and 85% ethanol for 30 minutes at room temperature and the fixed sample was incubated in PBS + 0.1% Triton X-100 for 2–5 minutes. The sample was given wash with PBS for 10 to 15 minutes. The fixed samples were further treated as described [52] with few modifications. Primary antibodies used were against CorA domain of MoMnr2. Secondary antibody, TRITC-conjugated anti-rabbit IgGs raised in goat, (Sigma Chemical Co, St Louis, MO, USA) diluted to 1:20 in PBS was used for staining for 2 hours. Three washes each of 15 minutes were given with PBST which was followed by vacuolar staining with Oregon green 488 carboxylic acid diacetate (cDFFDA) (Molecular Probes, Invitrogen, California, USA) for 10 minutes followed by three washes with PBST. The slides were observed under 63X using LSM 700 microscope (Carl Zeiss, Jena, Germany) at 557nm excitation and 576nm emission for TRITC and at 501nm excitation and 526nm emission for Oregon green 488. Image analysis was done using ZEN software.

### Plasmid construction and transformation of *M*. *oryzae*

The vector pGKO2-*MoALR2* was constructed for carrying out targeted disruption in WT. Full length gene of *MoALR2* (1.9kb) was PCR amplified, end filled and cloned at *EcoR*V site in pBluescript KS (+) to give KS-*MoALR2*. The HPT cassette used for disrupting the gene *MoALR2* was taken out from pAN7.1 [27] vector using *Bgl*II and *Hind*III having glyceraldehyde 3 phosphate dehydrogenase (gpdA) promoter, end filled and cloned at *EcoR*V site (present in between the *MoALR2* gene). The whole disruption cassette (~6kb) was moved from pBluescript KS (+) and cloned into a binary vector pGKO2 at *Kpn*I and *Spe*I site. The *A*. *tumefaciens* strain LBA4404/pSB1 was first transformed with pGKO2-*MoALR2-*HPT via triparental mating (Helper plasmid pRK2013). The transformed *Agrobacterium* was then used to carry out *A*. *tumefaciens* mediated transformation of *M*. *oryzae* as described [53]. The transformants were selected on Hygromycin (200μg ml^-1^) and 5-fluoro-2’-deoxyuridine (5μM). We attempted several rounds of transformation to disrupt *MoALR2* using ATMT. Protoplast transformation was also carried out with the full disruption cassette and by split marker technique (two different overlap regions). Further, supplementation of different Mg^2^ concentrations during selection was done to overcome the selective disadvantage facing slow growing mutants. A smaller disruption cassette of *MoALR2*(~4kb) was also made in the vector pBluescript KS (+) where KS-*MoALR2* was digested with *EcoR*V (site present in between the gene) and HPT cassette of 2kb was PCR amplified from pSilent and cloned at *EcoR*V site in KS-*MoALR2* to give pBSKS-*MoALR2*-HPT. This cassette was used to carry out transformation of the *Δku80* strain. Targeted deletion of *MoMNR2* was carried out by transforming WT protoplast with a knockout construct which was obtained by double-joint PCR [54], where ~1kb flanking sequence of *MoMNR2* was amplified and fused to Zeocin resistance cassette. Transformants were selected on Zeocin (300μg ml^-1^). They were screened with PCR using different sets of primer combinations and confirmed for target gene replacement by Southern blot analysis using both the flanking sequences as probes.

For knockdown of *MoALR2* a ~110 bp fragment of *MoALR2*, which spans the siRNA matching to the 5’ UTR, was amplified and cloned in pSilent-Dual 2 vector [5] at *Sma*I site. For simultaneous silencing of *MoALR2* and *MoMNR2*, the full length gene of *MoALR2* cloned in pBluescript KS (+) was digested with *Kpn*I and *BamH*I to give a fragment of 1.4kb (having a portion of the CorA domain) and was cloned in anti-sense orientation in pSilent-1 [55] at *Kpn*I and *Bgl*II site. For RNAi approach, the full length gene of *MoALR2* cloned in pBluescript KS (+) was digested with *Hind*III and 525bp fragment (spanning CorA domain) was cloned in pSilent-Dual 2 vector at *Hind*III site. The knockdown constructs were used for protoplast transformation of WT as described [31]. Putative knockdown transformants were selected on Hygromycin (200μg ml^-1^) and Geneticin (1mg ml^-1^). Untransformed WT was kept as a control which did not grow on either Hygromycin or Geneticin medium. Vector transformation (pSD2) was also done as a control. The transformants were maintained as monoconidial isolates to obtain pure cultures. The knockdown transformants were screened by PCR and confirmed by Southern hybridization.

### Nucleic acid manipulation and Southern Blotting

Fungal genomic DNA was extracted as described by Dellaporta et al. [56]. Southern blot analysis was carried out as previously described Sambrook et al. [57]. In case of *Δmnr2* and WT, genomic DNA was digested with *Hind*III, *EcoR*I and *EcoR*V and the blot was probed first with a 1kb upstream fragment of *MoMNR2* and then re-probed with a 1kb downstream fragment of *MoMNR2*. The knockdown transformants for *MoALR2* were digested with *EcoR*I and probed with 1.2kb upstream fragment of *MoALR2*. The simultaneously silenced transformants, A2 and A15 was digested with *Sal*I and the blot was probed with TrpC promoter (~400bp). The probes were labeled and hybridizing bands were detected using Gene Images AlkPhos Direct Labeling and Detection system as per manufacturer’s instructions (Amersham, Buckinghamshire, England).

### Quantitative Real Time PCR analysis for gene expression

Fungus was grown in Complete Medium (CM) for 72 hours (when no treatment was given to the biomass), else it was grown in CM for 48 hours, followed by two washes of the biomass with sterile milliQ water. The fungus was then transferred into Minimal Medium for 24 hours after which it was given treatments of Mg^2+^ or Ca^2+^ for the given time period. Fungal biomass was harvested and frozen in liquid nitrogen. Total RNA was isolated using TRIzol reagent (Invitrogen Life Technologies, California, USA). 2μg of total RNA was reverse transcribed into first strand cDNA using oligo (dT) primer and M-MuLV Reverse transcriptase (NEB, Massachusetts, USA). Quantitative PCR was performed by monitoring in real time the increase in fluorescence of the SYBR Green dye either on a Light Cycler system for real-time PCR (Roche Applied Science, Mannheim, Germany)or ABI 7900 HT real time PCR (Applied Biosystems, California, USA), according to the manufacturer’s instructions. Thermal cycling conditions consisted of 10 minutes at 95°C 1 cycle, followed by 40 cycles of 10 seconds at 95°C, 10 seconds at 55°C and 15 seconds at 72°C for SYBR chemistry. Also Taqman Probes (Applied Biosystems, California, USA) specific for gene(s) were used to validate and study expression profile quantitatively. Reaction conditions were according to manufacturer’s instructions. Thermal cycling conditions consisted of 2 minutes at 50°C 1 cycle, 10 minutes at 95°C 1 cycle, 15 seconds at 95°C and 1 minute at 60°C 40 cycles. Each qRT-PCR quantification was carried out in triplicate and every biological repeat was kept in duplicate. To compare the relative abundance of target gene transcripts, the average threshold cycle (Ct) was normalized to that of GPDH gene for each of the treated samples as2^-ΔCt^, where ΔCt = (Ct_gene_ of interest—Ct_GPDH_) and fold changes were calculated by 2^-ΔΔCt^, where ΔΔCt = (Ct _gene of interest_−Ct _GPDH_) _test condition_−(Ct_gene of interest_−Ct _GPDH_) _control_. The transcript levels were expressed as relative values, with 1 corresponding to the Wild type (WT).

#### Statistical Analyses

One-way ANOVA and non-parametric test was performed for all the statistical analyses wherein the mean of each column was compared with the mean of control column, followed by Fisher’s LSD test at 95% confidence interval. ** means P value at <0.0001 and * means significant at P value <0.05.

### X-Ray Fluorescence (XRF) for element analysis

For determining the levels of elements (mainly Mg^2+^ and Ca^2+^) in the knockout and knockdown transformants X- ray Fluorescence Analysis (XRF) [58] was performed. WT, knockout and knockdown transformants were grown in CM for 48 hours. The fungal biomass was washed twice with sterile milliQ water and the biomass was transferred into Minimal media for 24 hours. Then the biomass was grown under different concentrations of Mg^2+^ (4mM, 50mM and 250mM) for another 6 hours. The fungal biomass was harvested, frozen in liquid nitrogen and ground to fine powder. The biomass was dried completely at 37°C for 3–4 days, after which the element analysis was done using Energy dispersive X-ray Fluorescence Spectrometer EDX-720/800HS (Shimadzu, Singapore).

### Phenotypic Characterization of transformants

Vegetative growth of the knockdown and knockout transformants was measured on OMA and YEGA 5 days post inoculation. The experiments were performed with replicates in three independent experiments.

The ability to produce conidia was measured by counting the numbers of conidia for the knockdown and knockout transformants isolated from the surface. Quantification of conidia was done using a hemacytometer (Marienfeld Superior, Lauda-Konigshofen, Germany).

For appressorium formation, equal number of spores were inoculated on hydrophobic surface, Gelbond film (Amersham Pharmacia Biotech AB, Uppsala, Sweden) and incubated under moist conditions for 6 and 12 hours. For non-sporulating knockdown transformants, mycelial plugs from actively growing transformants (5^th^ day plate) were inoculated and incubated under moist conditions for 48 hours. Appressorium formation in the knockdown and knockout transformants was checked at 40X magnification (Olympus, Tokyo, Japan). The relative percentage of appressoria formed was calculated for each time interval.

For assaying the sensitivity of the knockdown and knockout transformants towards CorA specific inhibitor (Cobalt(III) hexaammine(Co(III)Hex), a concentration ranging from 300μM to 400μM of Cobalt(III) hexaammine was added to YEG medium. The sensitivity was assessed 5 days post inoculation and radial growth was measured for these transformants.

### Surface hydrophobicity assay

The knockout and knockdown transformants were tested for defects in surface hydrophobicity with water and detergent solution (0.02% SDS+5mM EDTA). 5 day old fungal culture grown on YEGA was inoculated with water and detergent solutions. The wettability of the transformants was checked by the extent to which water or detergent was retained on mycelia compared to WT.

### Western blotting

Total protein was extracted from WT and knockdown transformants grown in CM for 72 h (when no treatment was given to the biomass); else the fungus was grown in CM for 48 hours, which was followed by two washes of the biomass with sterile milliQ water. The fungus was then transferred into Minimal medium for 24 hours after which the fungus was given treatments of Mg^2+^ or Ca^2+^ in the present study for the given time period. Fungal biomass was harvested and frozen in liquid nitrogen. Protein was extracted in Urea Buffer (9.5M urea, 2% v/v NP40, 5% βME) for 1 hour at RT. The concentration of the protein was estimated by Bradford method. The protein samples were electrophoresed on 10% SDS–polyacrylamide gel, followed by electro transfer to PVDF membrane (Hybond ECL, Amersham, Buckinghamshire, England). The immunoblots were developed with 1° antibody against MoMnr2 CorA domain and 2° antibody conjugated with HRP using 3’-3’- diamino Benzidin tetrahydrochloride dehydrate (Fluka, Washington DC, USA) detection method (Bangalore Genei, Bangalore, India) and Super Signal West Pico Chemiluminescent substrate (Thermo Scientific, Rockford, USA) as per the manufacturer’s instructions.

### Cation sensitivity assay

For cation sensitivity assays, the knockout, knockdown transformants and WT were inoculated on YEGA having Zinc (Zn^2+^ 500μM), Cobalt (Co^2+^ 150μM), Manganese (Mn^2+^ 3mM), Iron (Fe^3+^ 2mM), Copper (Cu^2+^ 750μM), Aluminium (Al^3+^ 800μM), Nickel (Ni^2+^ 200μM). The sensitivity to different cations was studied by comparing the growth of transformants 5 days post inoculation with respect to WT. For all the sensitivity assays the minimum inhibitory concentration (MIC) was determined with respect to WT.

### Infection assay and cell wall integrity assay

Leaves of 21 day old rice seedlings of HR-12 cultivar were used for inoculating spores or mycelial plugs (for non-sporulating transformants) of knockout and knockdown transformants and were placed on water agar with kinetin (2 mg l^-1^). Disease symptoms were recorded after 3–4 days.

For the cell wall integrity assay, WT, knockout and knockdown transformants were inoculated on YEGA containing Congo Red (1.5mg ml^-1^and 2mg ml^-1^) or Caffeine (2.5mM and 3mM). Sensitivity to these cell wall stress molecules was studied by comparison of growth to that of WT 5 days post inoculation.

### Quantification of intracellular cAMP levels

WT, knockout and knockdown transformants were grown in CM for 48 hours. The fungal biomass was washed twice with sterile milliQ water and then the biomass was transferred into Minimal media containing 4mM Mg^2+^ for 24 hours. The biomass was harvested and frozen. The fungal biomass was ground to fine powder in liquid nitrogen and after the liquid nitrogen was evaporated, equal weight of frozen biomass (0.1gm) was taken and homogenized in 300μl of 0.1N HCl. The sample was vortexed for 30 minutes, followed by centrifugation at 5000 x g for 15 minutes at room temperature. The supernatant was collected and 100μl of sample in each case was taken. Quantification of intracellular cAMP levels was carried out by cAMP Direct Immunoassay Kit as per manufacturer’s instructions (Calbiochem, Darmstadt, Germany).

## Supporting Information

S1 Fig Southern blot analysis of *Δmnr2* and WT. (A) Schematic representation of *MoMNR2* locus and *MoMNR2* knockout cassette. (B) Wild type (WT) and three independent transformants (T1, T2, T3) for *Δmnr2* were digested with three different restriction enzymes and the blot was probed with two different probes to confirm targeted replacement of *MoMNR2* (L- 1Kb ladder, P- Positive control).
(TIF)

S2 Fig Southern blot and Western blot analysis of knockdown transformants. (A) WT, pSD2 transformants and knockdown transformants both in the background of WT and *Δmnr2* were digested with *EcoR*I and probed with 1.2Kb fragment upstream to *MoALR2* to confirm integration of the silencing cassette. WT and simultaneously silenced transformants, A2 and A15, were digested with *Sal*I and probed with TrpCP to confirm integration of silencing construct. (B) Western blot showing levels of MoAlr2 and MoMnr2 proteins in WT and knockdown transformants using polyclonal antibodies raised against the CorA domain of MoMnr2. 30μg of protein was run on 10% SDS-PAGE and the blot was developed using luminal/enhancer + peroxide solution.
(TIF)

S3 Fig Element analysis and colony growth of knockdown transformants. (A) Intracellular levels of Zn^2+^ in WT, A2 and A15 were estimated by XRF. The values are expressed as relative values, with 1 corresponding to the WT at 4mM Mg^2+^. (B) Colony growth and melanization of WT, *Δmnr2* and knockdown transformants on OMA. Photographs were taken 9 days post inoculation.
(TIF)

S4 Fig Growth of WT under Mg^2+^ limiting conditions (EDTA). (A) Growth of WT in presence of different concentrations of EDTA. 3x3 mm mycelial plugs were inoculated on OMA with and without EDTA and growth was assessed 5 days post inoculation. (B) Restoration of growth on Mg^2+^ supplements in presence of EDTA (top). Growth of sectored colonies obtained under stress conditions (EDTA) (bottom). Growth of different sectors was assessed on OMA.
(TIF)

S5 Fig Sporulation and Appressorium formation in WT under Mg^2+^ limiting conditions (EDTA). (A) The ability of WT to sporulate was checked on OMA with different concentrations of EDTA 8 days post inoculation and quantified. (B) The ability to form appressoria in water, 0.25mM EDTA and 0.25mM EDTA+50mM Mg^2+^ was observed at different time intervals in WT and percentages of spores (ungerminated), germ tubes and appressoria formed were calculated for each time interval and for each condition.
(TIF)

S6 Fig Recovery of growth on Mg^2+^ supplements in double knockdown transformants. YEG and YEG with Congo Red (1.5mg/ml) and Caffeine (2.5mM) were supplemented with different concentrations of Magnesium. 2X2 mm mycelial plugs of WT and knockdown transformants A2 and A15 were inoculated. Recovery in growth was assessed 5 days post inoculation.
(TIF)

S7 Fig Growth of WT and *Δmnr2* on media supplemented with EDTA. WT and *Δmnr2* were grown on OMA and YEGA supplemented with 0.5mM EDTA. Growth was assessed 5dpi. *Δmnr2* shows more growth inhibition than WT.
(TIF)

S1 Table Disruption of *MoALR2* by different approaches. Table shows number of transformants obtained from ATMT and protoplast transformation (with full cassette and split marker using two different lengths of overlaps) and by using F2DU, different concentrations of MgSO_4_ and Co(III)Hex. for selection.
(DOCX)

S2 Table Relative Expression of CorA Mg^2+^ transporters, *MoALR2* and *MoMNR2*, in knockdown transformants. (DOCX)

S3 Table Vegetative Growth of WT, *Δmnr2* and knockdown transformants. Vegetative growth was measured on OMA 5 days post inoculation. Data are presented as mean±SD from three independent experiments.
(DOCX)

S4 Table List of primers used in the present study. (DOCX)
