# Supplementary figures and images for: Magnesium Uptake by CorA Transporters Is Essential for Growth, Development and Infection in the Rice Blast Fungus Magnaporthe oryzae
Source: PLoS One. 2016 Jul 14;11(7):e0159244. doi: 10.1371/journal.pone.0159244 (PMC4945025; doi:10.1371/journal.pone.0159244)

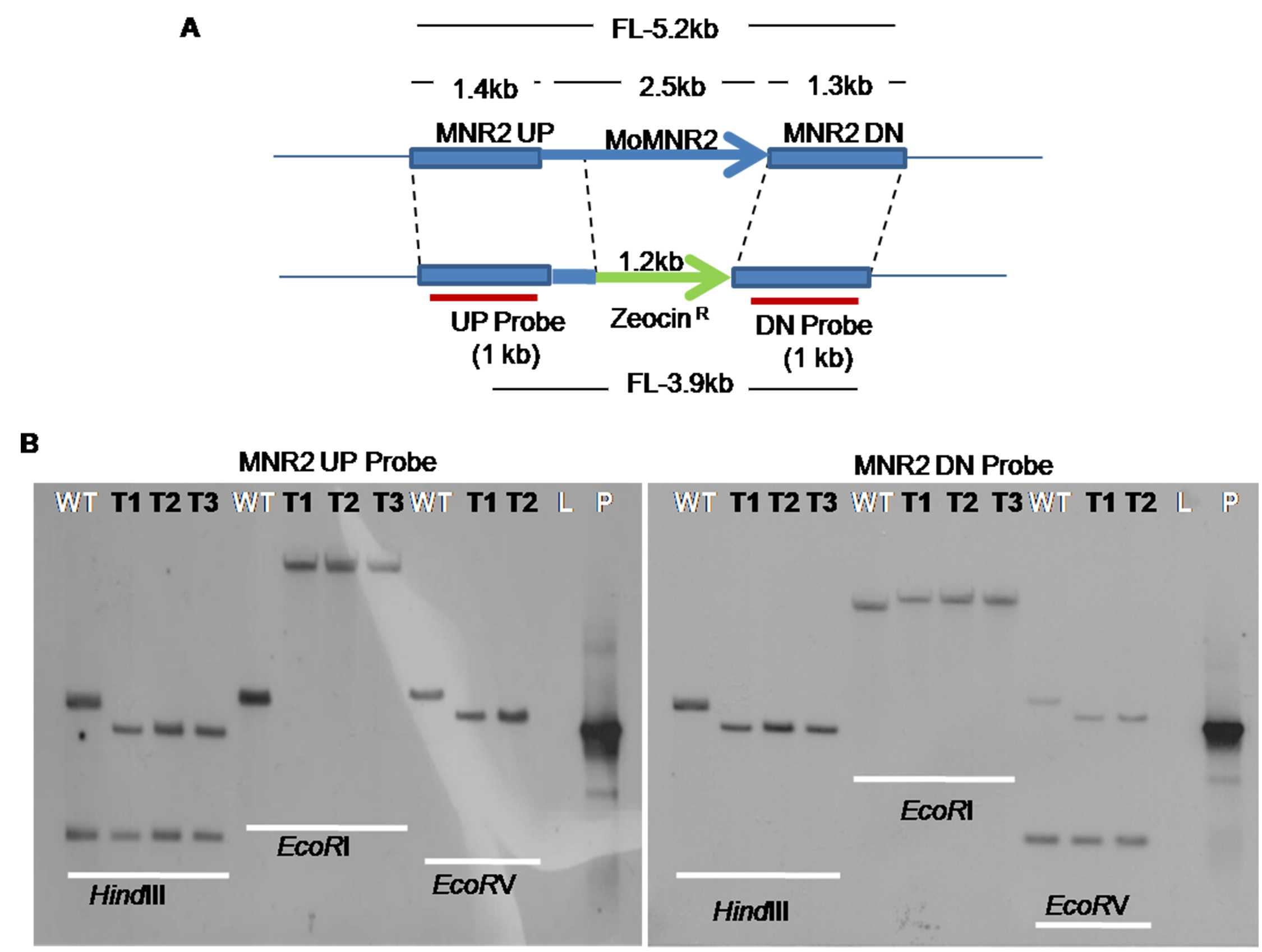

Supplement: S1 Fig — (A) Schematic representation of MoMNR2 locus and MoMNR2 knockout cassette. (B) Wild type (WT) and three independent transformants (T1, T2, T3) for Δmnr2 were digested with three different restriction enzymes and the blot was probed with two different probes to confirm targeted replacement of MoMNR2 (L- 1Kb ladder, P- Positive control). (TIF) [file pone.0159244.s001.tif]

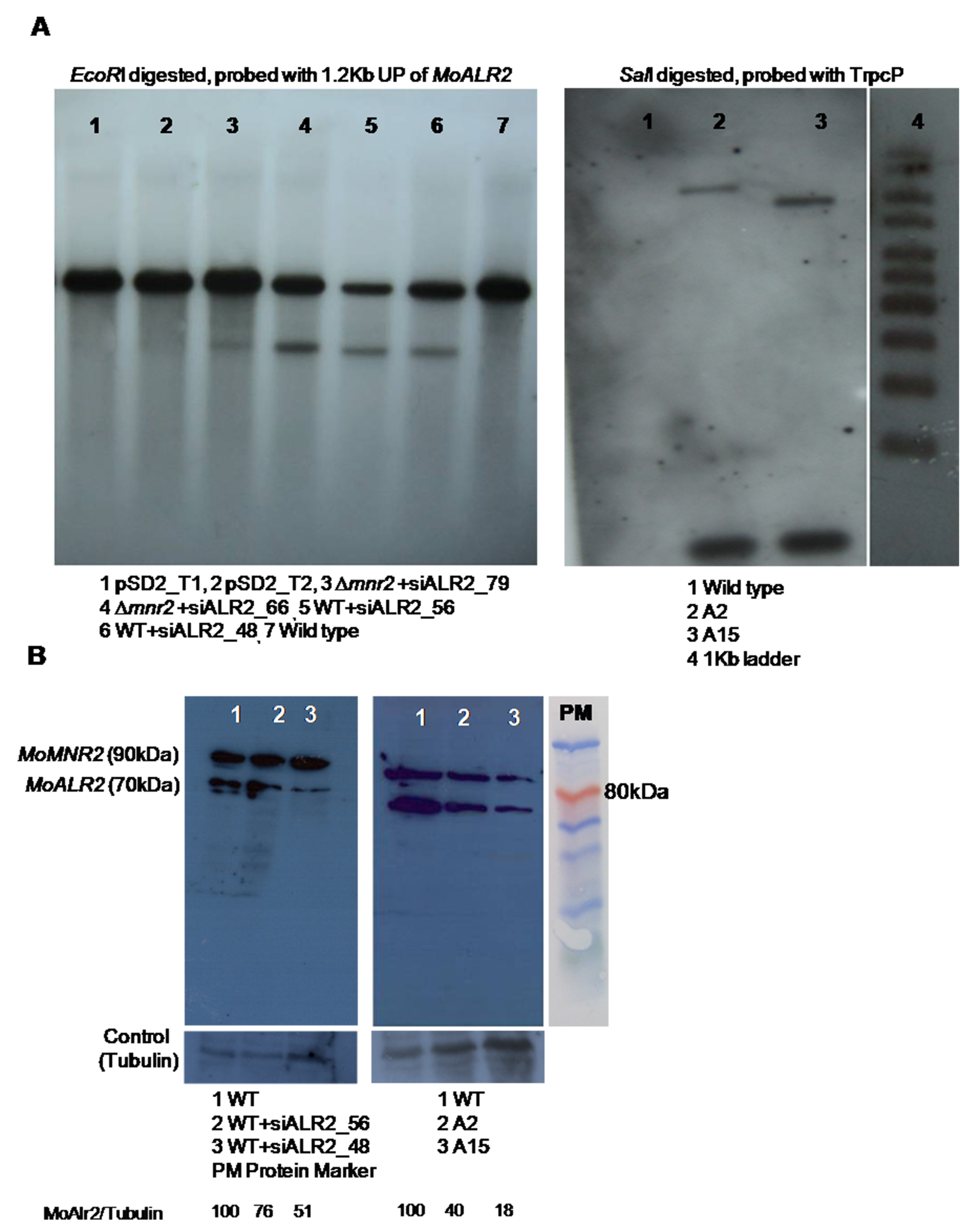

Supplement: S2 Fig — (A) WT, pSD2 transformants and knockdown transformants both in the background of WT and Δmnr2 were digested with EcoRI and probed with 1.2Kb fragment upstream to MoALR2 to confirm integration of the silencing cassette. WT and simultaneously silenced transformants, A2 and A15, were digested with SalI and probed with TrpCP to confirm integration of silencing construct. (B) Western blot showing levels of MoAlr2 and MoMnr2 proteins in WT and knockdown transformants using polyclonal antibodies raised against the CorA domain of MoMnr2. 30μg of protein was run on 10% SDS-PAGE and the blot was developed using luminal/enhancer + peroxide solution. (TIF) [file pone.0159244.s002.tif]

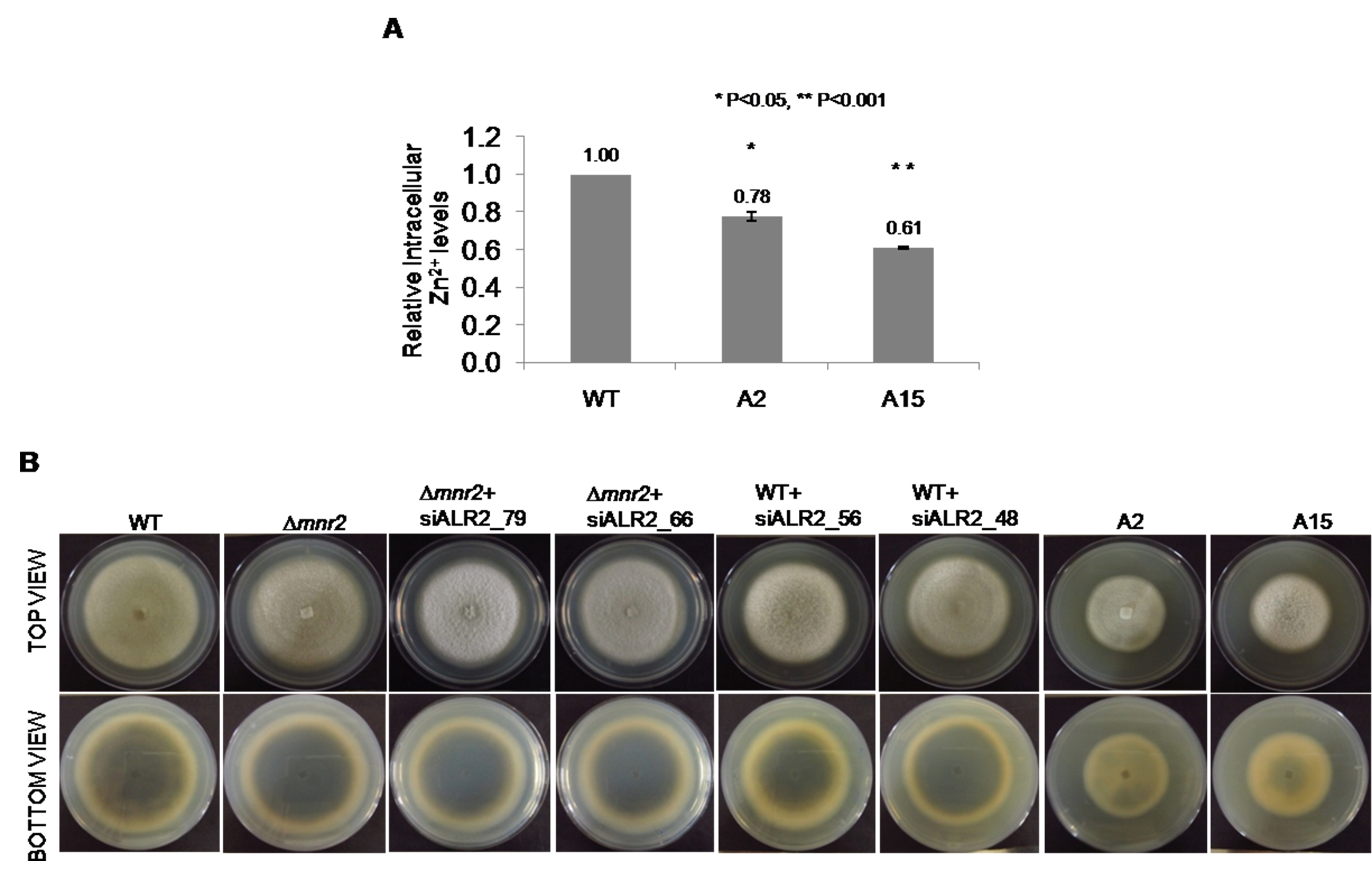

Supplement: S3 Fig — (A) Intracellular levels of Zn2+ in WT, A2 and A15 were estimated by XRF. The values are expressed as relative values, with 1 corresponding to the WT at 4mM Mg2+. (B) Colony growth and melanization of WT, Δmnr2 and knockdown transformants on OMA. Photographs were taken 9 days post inoculation. (TIF) [file pone.0159244.s003.tif]

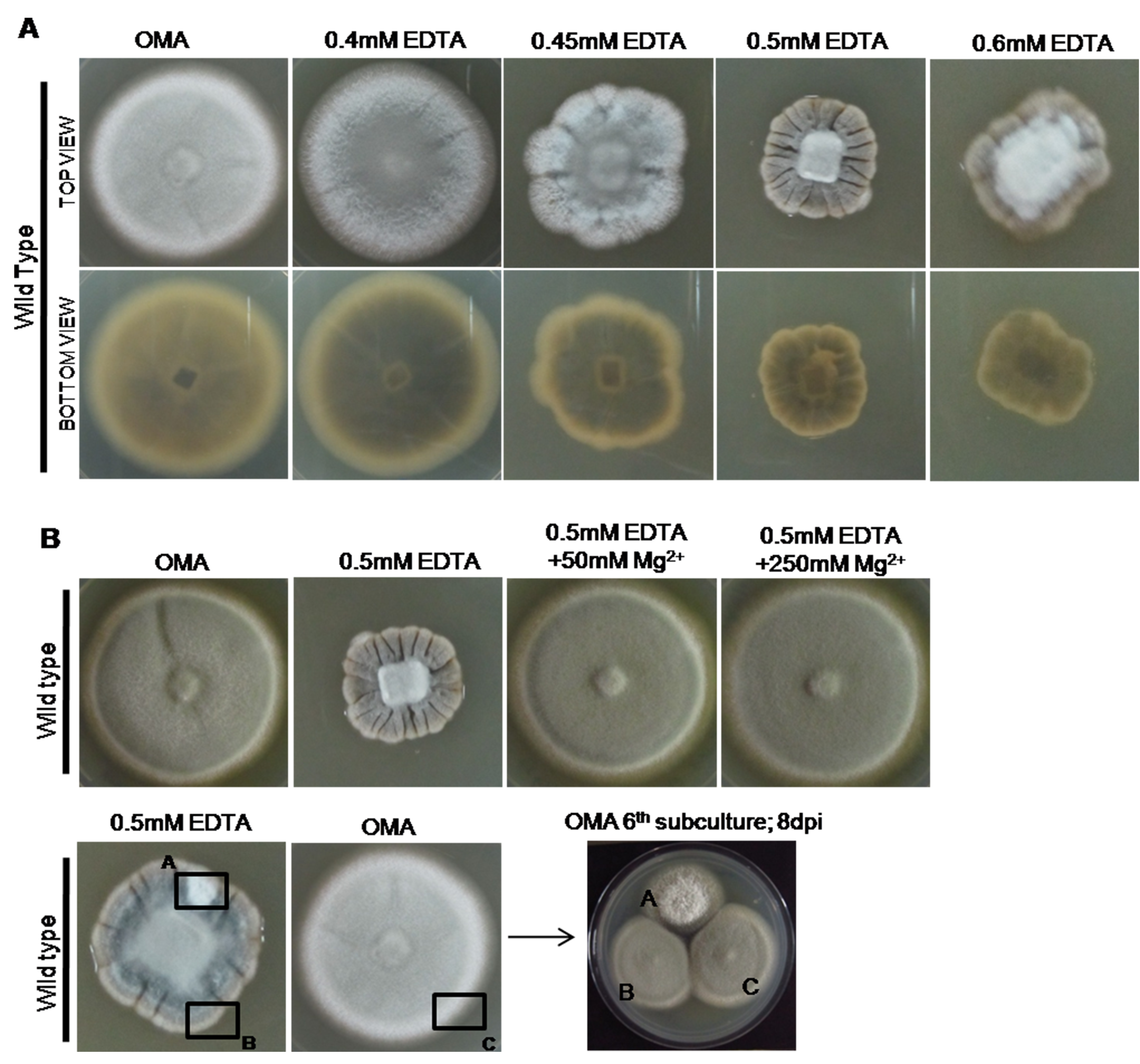

Supplement: S4 Fig — (A) Growth of WT in presence of different concentrations of EDTA. 3x3 mm mycelial plugs were inoculated on OMA with and without EDTA and growth was assessed 5 days post inoculation. (B) Restoration of growth on Mg2+ supplements in presence of EDTA (top). Growth of sectored colonies obtained under stress conditions (EDTA) (bottom). Growth of different sectors was assessed on OMA. (TIF) [file pone.0159244.s004.tif]

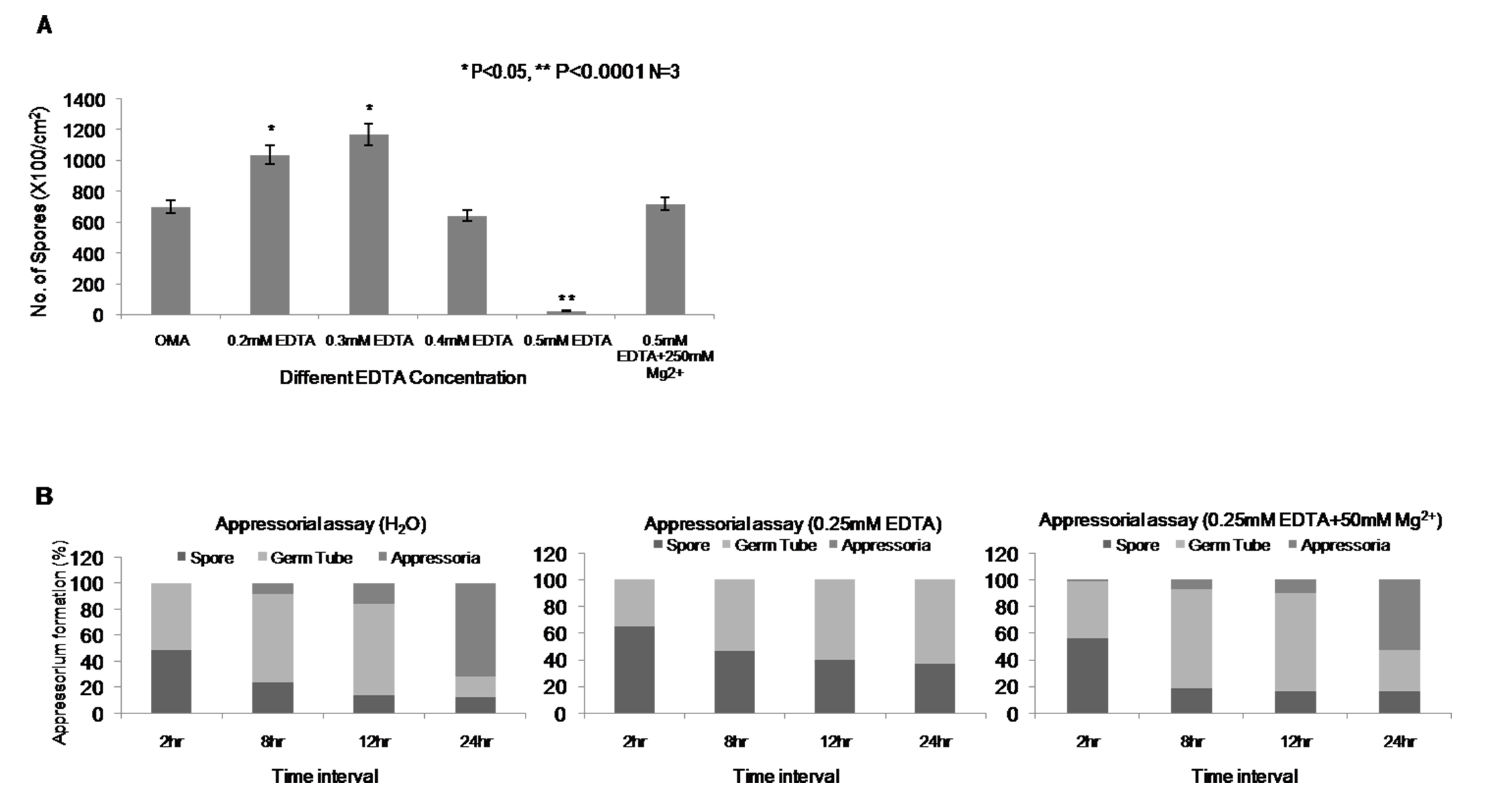

Supplement: S5 Fig — (A) The ability of WT to sporulate was checked on OMA with different concentrations of EDTA 8 days post inoculation and quantified. (B) The ability to form appressoria in water, 0.25mM EDTA and 0.25mM EDTA+50mM Mg2+ was observed at different time intervals in WT and percentages of spores (ungerminated), germ tubes and appressoria formed were calculated for each time interval and for each condition. (TIF) [file pone.0159244.s005.tif]

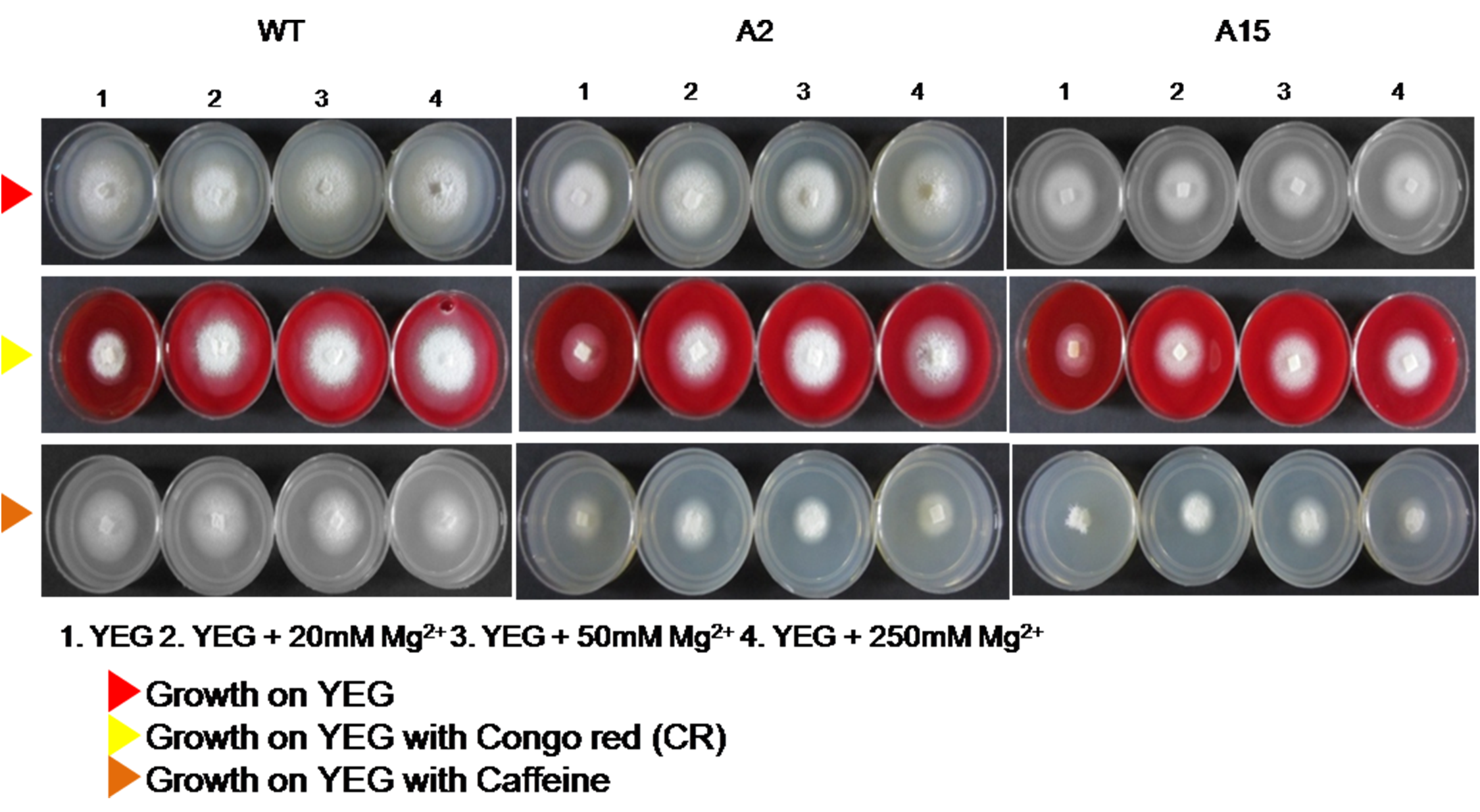

Supplement: S6 Fig — YEG and YEG with Congo Red (1.5mg/ml) and Caffeine (2.5mM) were supplemented with different concentrations of Magnesium. 2X2 mm mycelial plugs of WT and knockdown transformants A2 and A15 were inoculated. Recovery in growth was assessed 5 days post inoculation. (TIF) [file pone.0159244.s006.tif]

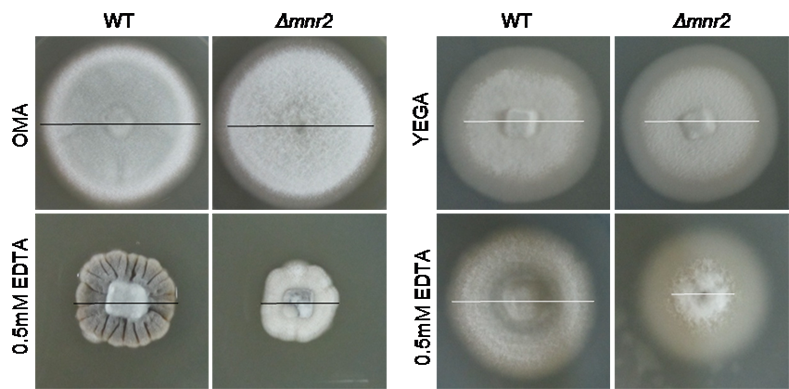

Supplement: S7 Fig — WT and Δmnr2 were grown on OMA and YEGA supplemented with 0.5mM EDTA. Growth was assessed 5dpi. Δmnr2 shows more growth inhibition than WT. (TIF) [file pone.0159244.s007.tif]
