# Supplementary material for: Magnesium Uptake by CorA Transporters Is Essential for Growth, Development and Infection in the Rice Blast Fungus Magnaporthe oryzae
Source: PLoS One. 2016 Jul 14;11(7):e0159244. doi: 10.1371/journal.pone.0159244 (PMC4945025; doi:10.1371/journal.pone.0159244)
Supplement: S1 Table — Table shows number of transformants obtained from ATMT and protoplast transformation (with full cassette and split marker using two different lengths of overlaps) and by using F2DU, different concentrations of MgSO4 and Co(III)Hex. for selection. (DOCX) [file pone.0159244.s008.docx]

|  | **No. of transformants** | **No. of transformants growing on F2DU (5µM)** | **Concentration of MgSO_4_ used** | **Co (III)Hex. used for secondary selection** | **True Disruptants obtained** |
| --- | --- | --- | --- | --- | --- |
| **ATMT** **in wild type B157** | 202 | 20 | 100mM | N.A. | NONE |
| **Protoplast transformation with Disruption cassette** **in wild type B157** | 38 | N.A. | 10mM/100mM | 350µM/400µM | NONE |
| **Protoplast transformation with Split Marker (overlap of 1.1Kb)** **in wild type B157** | 55 | N.A. | 10mM/100mM | 350µM/400µM | NONE |
| **Protoplast transformation with Split Marker (overlap of 400bp)** **in wild type B157** | 44 | N.A. | 10mM/100mM | 350µM/400µM | NONE |
| **Protoplast transformation with KS-MoALR2-HPT (~4Kb) disruption cassette in *Δku80* strain** | 34 | N.A. | 100mM | N.A. | NONE |
