# Supplementary material for: Magnesium Uptake by CorA Transporters Is Essential for Growth, Development and Infection in the Rice Blast Fungus Magnaporthe oryzae
Source: PLoS One. 2016 Jul 14;11(7):e0159244. doi: 10.1371/journal.pone.0159244 (PMC4945025; doi:10.1371/journal.pone.0159244)
Supplement: S2 Table — (DOCX) [file pone.0159244.s009.docx]

| **Knockdown transformants** | **Relative Expression of *MoALR2* (%)** | **Relative Expression of *MoMNR2* (%)** |
| --- | --- | --- |
| RNAi knockdown transformants in WT | | |
| R1 | 43 | 64 |
| R2 | 54 | 62 |
| R3 | 60 | 67 |
| R5 | 57 | 81 |
| R10 | 68 | 83 |
| R19 | 55 | 76 |
| Anti-sense knockdown transformants in WT | | |
| A2 | 43 | 52 |
| A3 | 54 | 57 |
| A4 | 65 | 59 |
| A10 | 47 | 60 |
| A13 | 73 | 54 |
| A15 | 30 | 37 |
| A19 | 80 | 90 |
| *MoALR2* silencing in WT | | |
| WT_1 | 85 | 93 |
| WT_4 | 81 | 90 |
| WT_5 | 48 | 89 |
| WT_6 | 56 | 90 |
| WT_8 | 75 | 87 |
| WT_9 | 78 | 94 |
| *MoALR2* silencing in *Δmnr2* | | |
| *Δmnr2*_3 | 88 | 0 |
| *Δmnr2*_4 | 83 | 0 |
| *Δmnr2*_7 | 81 | 0 |
| *Δmnr2*_9 | 86 | 0 |
| *Δmnr2*_10 | 80 | 0 |
| *Δmnr2*_18 | 78 | 0 |
| *Δmnr2*_20 | 66 | 0 |
