# Supplementary material for: Magnesium Uptake by CorA Transporters Is Essential for Growth, Development and Infection in the Rice Blast Fungus Magnaporthe oryzae
Source: PLoS One. 2016 Jul 14;11(7):e0159244. doi: 10.1371/journal.pone.0159244 (PMC4945025; doi:10.1371/journal.pone.0159244)
Supplement: S3 Table — Vegetative growth was measured on OMA 5 days post inoculation. Data are presented as mean±SD from three independent experiments. (DOCX) [file pone.0159244.s010.docx]

| **Transformants** | **Growth diameter (centimeters)** |
| --- | --- |
| WT | 6.00±0.06 |
| *Δmnr2* | 5.70±0.11 |
| *Δmnr2*+siALR2_79 | 5.67±0.04 |
| *Δmnr2*+siALR2_66 | 5.43±0.07 |
| WT+siALR2_56 | 5.40±0.06 |
| WT+siALR2_48 | 5.27±0.04 |
| A2 | 4.70±0.06 |
| A15 | 4.53±0.04 |
