# Supplementary material for: Magnesium Uptake by CorA Transporters Is Essential for Growth, Development and Infection in the Rice Blast Fungus Magnaporthe oryzae
Source: PLoS One. 2016 Jul 14;11(7):e0159244. doi: 10.1371/journal.pone.0159244 (PMC4945025; doi:10.1371/journal.pone.0159244)
Supplement: S4 Table — (DOCX) [file pone.0159244.s011.docx]

| **S.No.** | **Primer Name** | **Sequence (5’-3’)** | **Application** |
| --- | --- | --- | --- |
| 1 | ALR2_F | ATGTCTGACCACGACGAACACG | *ALR2* ORF Forward primer |
| 2 | ALR2_R | TAAGCTTCCTCGCAGTGCAAAG | *ALR2* ORF Reverse primer |
| 3 | PMoMNR2_F (NdeI) | CAACATATGGAGCGCCCAGTCTGGTGGC | *MNR2* CorA domain Forward |
| 4 | PMoMNR2_R (KpnI) | ACCGGTACCTTCCCATACAATTTCTTGACTAGC | *MNR2* CorA domain Reverse |
| 5 | MNR2_F | ATGGAGGCTTCCAGTTGCCAC | *MNR2* ORF Forward primer |
| 6 | MNR2_R | TAGCCCATACAATTTCTTGAC | *MNR2* ORF Reverse primer |
| 7 | qMAC1_F | AGGATTGACGATGATTTGAACC | *MoMAC1* qPCR Forward primer |
| 8 | qMAC1_R | GGCGAGGTCCCTTCGATAG | *MoMAC1* qPCR Reverse primer |
| 9 | siRNA ALR2_F | AAGAGCTCTCCCATCACGC | *MoALR2* 5’UTR Forward primer |
| 10 | siRNA ALR2_R | GAATAGAATTCTTTCAGGGGTC | *MoALR2* 5’UTR Reverse primer |
| 11 | MNR2 Up_F | GAGAGGGCGGGGCATTTTGC | *MNR2* 5’ flank Forward primer |
| 12 | MNR2 Up_R | ctccttcaatatcagttaacgtcCGGTGAGTTCTCTTGCGCCG | *MNR2* 5’ flank Reverse primer |
| 13 | MNR2 Dn_F | gaaaattccgtcaccagccctggCGGATTGGATCTCTTACGCC | *MNR2* 3’ flank Forward primer |
| 14 | MNR2 Dn_R | GGAAGCTTGAGTGAGCATGG | *MNR2* 3’ flank Reverse primer |
| 15 | MNR2 Nest_F | CTCAGCCCAAAAATCATCAGC | Nested Forward primer for *MNR2* |
| 16 | MNR2 Nest_R | TGTTTCAAAAATGCCTTCCCACC | Nested Reverse primer for *MNR2* |
| 17 | TrpCP_F (KpnI) | AGCGGTACCGACGTTAACTGATATTGAAG | 5’ TrpC Promoter Amplification |
| 18 | TrpCT_R (BamHI) | TCAGGATCCAACCCAGGGCAGGTGACGGA | 3’ TrpC Terminator Amplification |
| 19 | HPT 500bp_F | ATGTCCTGCGGGTAAATAGC | hygromycin cassette Forward |
| 20 | HPT 500bp_R | TGTTATGCGGCCATTGTCCG | hygromycin cassette Reverse |
| 21 | MGG_06358_F | CTTCTCCCTCGGTCTCAGTTC | *MoABP1* qPCR Forward primer |
| 22 | MGG_06358_R | CTTGGTCGGCAAAGGTACGA | *MoABP1* qPCR Reverse primer |
| 23 | MGG_06358_M | CCGCACCGCCTCCAGT | Taqman Probe for *MoABP1* |
| 24 | MGG_04116_F | CCATCTCAATCCGCCCAATCTC | *MoMTI1* qPCR Forward primer |
| 25 | MGG_04116_R | CGACTTCACACCTGCAATTGGA | *MoMTI1* qPCR Reverse primer |
| 26 | MGG_04116_M | CTCGTCCGCCTATGCC | Taqman Probe for *MoMTI1* |
| 27 | MGG_05133_F | TCCTCCGAGGCCTTTCCT | *MoCRZ1* qPCR Forward primer |
| 28 | MGG_05133_R | GCGCATAATCGATGTTGATTGTCG | *MoCRZ1* qPCR Reverse primer |
| 29 | MGG_05133_M | ATCTGCGCCAAGCCAT | Taqman Probe for *MoCRZ1* |
| 30 | MGG_00865_F | CATCGACCACGTTCAGAAGCT | *MoFKS1* qPCR Forward primer |
| 31 | MGG_00865_R | GAACGTGGGAGCCCTAAGAG | *MoFKS1* qPCR Reverse primer |
| 32 | MGG_00865_M | TCCGCTTGCCCTCCTG | Taqman Probe for *MoFKS1* |
| 33 | MGG_01802_F | TGCGTTCGGATACGTTAGTGT | *MoCHS1* qPCR Forward primer |
| 34 | MGG_01802_R | GCACGGAAGCGGTATGC | *MoCHS1* qPCR Reverse primer |
| 35 | MGG_01802_M | TTGCCCGGTGCTTTC | Taqman Probe for *MoCHS1* |
| 36 | MGG_09962_F | CAGGCCTCAGGTGGTTACATG | *MoCHS4* qPCR Forward primer |
| 37 | MGG_09962_R | TTGTGCGACTCTCGTTGGT | *MoCHS4* qPCR Reverse primer |
| 38 | MGG_09962_M | CCCAGCTCCACAGCCT | Taqman Probe for *MoCHS4* |
| 39 | MGG_04943_F | CGATTGATGTATGGTCGGTTGGAT | *MoSPM1* qPCR Forward primer |
| 40 | MGG_04943_R | CGTAATCGCGACCCTTGAAGAA | *MoSPM1* qPCR Reverse primer |
| 41 | MGG_04943_M | CTCTGCCAAAATGC | Taqman Probe for *MoSPM1* |
| 42 | MGG_06482_F | ACGGTATTCGCCTTAAAGGTGATC | *MoMKK1* qPCR Forward primer |
| 43 | MGG_06482_R | CGCATGATCTGCTTCTTCACATC | *MoMKK1* qPCR Reverse primer |
| 44 | MGG_06482_M | CCAACCCAGACCCC | Taqman Probe for *MoMKK1* |
| 45 | MGG_00883_F | GACAGGCCAAAGAAGGGTTCA | *MoMAPK3* qPCR Forward primer |
| 46 | MGG_00883_R | GAGGTACACGCTGAGAGGGATA | *MoMAPK3* qPCR Reverse primer |
| 47 | MGG_00883_M | ACCGTCCATACTTCCC | Taqman Probe for *MoMAPK3* |
| 48 | MGG_08843_F | CTTGATTCTTCCTGGCGAGGATAT | *MoALR2* qPCR Forward primer |
| 49 | MGG_08843_R | TCGGCTGTTGGGTTGCT | *MoALR2* qPCR Reverse primer |
| 50 | MGG_08843_M | TCGAGCCACCAGACTC | Taqman Probe for *MoALR2* |
| 51 | MGG_09884_F | CCGAGTCCGACAATGATGATATGAC | *MoMNR2* qPCR Forward primer |
| 52 | MGG_09884_R | TGGATTGAAGGAGGTACTGAAGTCT | *MoMNR2* qPCR Reverse primer |
| 53 | MGG_09884_M | CCGTCAACCAACTCC | Taqman Probe for *MoMNR2* |
| 54 | MGG_07176_F | GACTCTCACGTTATCCTGATTTGCT | *MoRHO1* qPCR Forward primer |
| 55 | MGG_07176_R | CCACTTCTCTTGGACGTTGTCAA | *MoRHO1* qPCR Reverse primer |
| 56 | MGG_07176_M | TCGCCATCGACTCTCC | Taqman Probe for *MoRHO1* |
| 57 | MGG_03147_F | TCCTCCCGGGCTTCTACAG | *MoGPDH* qPCR Forward primer |
| 58 | MGG_03147_R | CTGAGGTCTGCGGGTCAA | *MoGPDH* qPCR Reverse primer |
| 59 | MGG_03147_M | TCAGGCCCATGTTTGC | Taqman Probe for *MoGPDH* |
| 60 | MGG_10315_F | CAACAGCAAGGAGCTTAAGAACTC | *MoMPG1* qPCR Forward primer |
| 61 | MGG_10315_R | GCCGCTGAGAACGTCGAT | *MoMPG1* qPCR Reverse primer |
| 62 | MGG_10315_M | TCGGCACCAGACTTG | Taqman Probe for *MoMPG1* |
| 63 | MGG_01173_F | GCTGTGCGACCGACATC | *MoMPG2* qPCR Forward primer |
| 64 | MGG_01173_R | GAGGGCTGGCCACAGT | *MoMPG2* qPCR Reverse primer |
| 65 | MGG_01173_M | TCGGCCTCGCCAACCT | Taqman Probe for *MoMPG2* |
